# Inhibition is a prevalent mode of activity in the neocortex around awake hippocampal ripples in mice

**DOI:** 10.7554/eLife.79513

**Published:** 2023-01-16

**Authors:** Javad Karimi Abadchi, Zahra Rezaei, Thomas Knöpfel, Bruce L McNaughton, Majid H Mohajerani

**Affiliations:** 1 Canadian Centre for Behavioral Neuroscience, University of Lethbridge Lethbridge Canada; 2 Laboratory for Neuronal Circuit Dynamics, Imperial College London London United Kingdom; 3 Department of Physics, Hong Kong Baptist University Kowloon Tong Hong Kong; 4 Department of Neurobiology and Behavior, University of California Irvine United States; McGill University Canada; University of Texas at Austin United States

**Keywords:** hippocampus, neocortex, hippocampal-neocortical interaction, memory consolidation, sharp-wave ripple, voltage/glutamate/calcium imaging, Mouse

## Abstract

Coordinated peri-ripple activity in the hippocampal-neocortical network is essential for mnemonic information processing in the brain. Hippocampal ripples likely serve different functions in sleep and awake states. Thus, the corresponding neocortical activity patterns may differ in important ways. We addressed this possibility by conducting voltage and glutamate wide-field imaging of the neocortex with concurrent hippocampal electrophysiology in awake mice. Contrary to our previously published sleep results, deactivation and activation were dominant in post-ripple neocortical voltage and glutamate activity, respectively, especially in the agranular retrosplenial cortex (aRSC). Additionally, the spiking activity of aRSC neurons, estimated by two-photon calcium imaging, revealed the existence of two subpopulations of excitatory neurons with opposite peri-ripple modulation patterns: one increases and the other decreases firing rate. These differences in peri-ripple spatiotemporal patterns of neocortical activity in sleep versus awake states might underlie the reported differences in the function of sleep versus awake ripples.

## Introduction

Hippocampal-neocortical interactions around hippocampal ripples play an important role in memory processes (Buzsáki, 2015; Buzsáki, 1989; Qin et al., 1997; Schwindel and McNaughton, 2011). The functional role of such interactions is believed to be brain state-dependent such that they are involved in memory consolidation during non-rapid eye movement (NREM) sleep, while they are implicated in memory-guided behavior such as planning and memory retrieval in waking state (Ólafsdóttir et al., 2018; Roumis and Frank, 2015; Tang and Jadhav, 2019). This state-dependent functional dichotomy poses a question: how does spatiotemporal dynamics of hippocampal-neocortical network interactions differ in the two states?

There are pronounced differences in the neocortical activity patterns between NREM sleep and quite wakefulness. The most prominent difference is the near absence of so-called slow-oscillations (SO) during wakefulness. SO, a quasi-synchronous ≤ 1 Hz rhythmic fluctuation observed in local-field potential (LFP) and EEG recordings throughout the neocortex during NREM sleep, is partly correlated with hippocampal ripples, and recent memory reactivation in cortex is strongly locked to ripples (Benchenane et al., 2010; Kaefer et al., 2020; Peyrache et al., 2009; Rothschild et al., 2016). However, given the near absence of SO in wakefulness (Tang et al., 2017), it is not straightforward to extrapolate from sleep to wakefulness, although a few studies have shown that the proportion of neurons whose spiking activity is suppressed around hippocampal ripples is significantly higher in wakefulness compared with sleep (Jadhav et al., 2016; Tang et al., 2017).

In the present study, we extended the previous results by imaging the activity of a large portion of the dorsal neocortical mantle in awake mice, with concurrent LFP and multi-unit activity (MUA) recording from the pyramidal layer of the dorsal CA1. Wide-field glutamate and voltage recording were used to capture the excitatory synaptic input and the membrane potential fluctuations across neocortical regions and to correlate them with the occurrence of hippocampal ripples. A sharp contrast in the peri-ripple neocortical activity between the awake and sleep states was observed. To further elaborate on this contrast, we used the two-photon calcium imaging to focus on the agranular retrosplenial cortex (aRSC) whose glutamate and voltage activity patterns were different from the rest of the imaged regions. Our results suggest that inhibition is more pronounced in peri-ripple neocortical activity in awake than sleep states.

## Results

**Figure 1.**
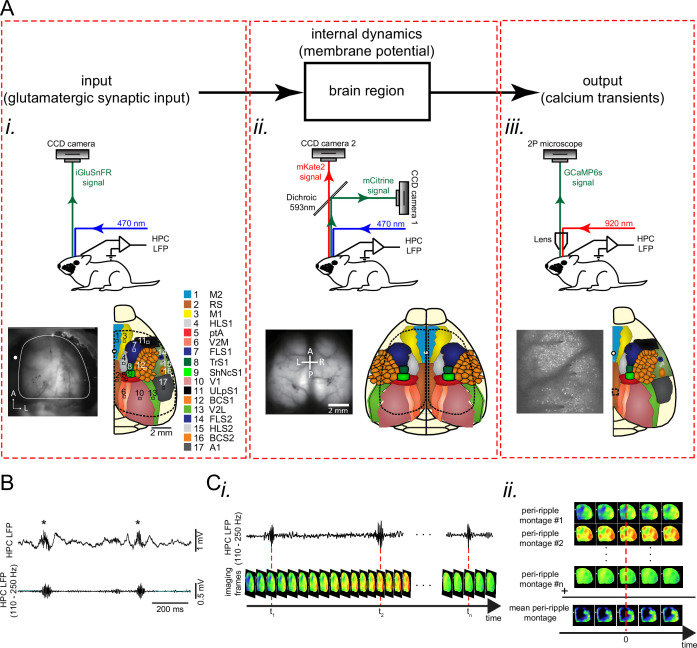
Experimental protocol for investigating peri-ripple neocortical activity during the awake state. (**A**) Top: each region could be modeled as an input-output block with internal dynamics. Bottom (i-iii): experimental setups, exemplar imaging windows, and schematic of the regions included in the windows for unilateral wide-field glutamate imaging (**i**), bilateral wide-field voltage imaging (ii), and two-photon calcium imaging (iii) which were conducted for monitoring input, internal dynamics, and output, respectively. (**B**) Top: an exemplar local field potential (LFP) trace recorded from the pyramidal layer of the CA1 subfield of the dorsal hippocampus. Asterisks denote detected ripples. Bottom: ripple-band (110–250 Hz) filtered version of the top trace. (**C**) Schematic of peri-ripple (ripple-triggered) averaging analysis. (**i**) Schematic of concurrently recorded LFP and imaging signals. Red dashed lines indicate the timestamp of the center of detected ripples. (ii) The imaging frames around the timestamp of the detected ripples are aligned with respect to the ripple centers and averaged. This figure has one figure supplement.

To study peri-ripple activity across neocortical regions in the awake state, we utilized three imaging modalities to shed light on different aspects of the problem at hand. First, to capture the internal dynamics of neocortical regions, wide-field voltage imaging with voltage indicator (butterfly1.2; voltage-sensitive fluorescent protein [VSFP] mice) expressed in the excitatory neurons of the neocortical layers II/III was used (Figure 1Aii). Second, to capture the excitatory input to the neocortical regions, wide-field imaging with intensity-based glutamate-sensing fluorescent reporter (iGluSnFR; iGlu-Ras mice) indicator expressed in the excitatory neurons of the neocortical layers II/III was used (Figure 1Ai). Last, to estimate the spiking output of neocortical neurons, two-photon calcium imaging of the aRSC superficial layers in Thy1-GCamp mice was conducted (Figure 1Aiii). In addition, to compare the peri-ripple glutamatergic transmission in superficial versus deep neocortical layers, wide-field imaging of iGluSnFR activity in all neocortical layers (iGlu-EMX mice) was conducted. In all the imaging modality experiments, concurrent LFP and MUA recordings from pyramidal layer of the CA1 subfield of the dorsal hippocampus was performed, and the hippocampal LFP was used to detect ripples (Figure 1B). Moreover, in three out of four sets of the experiments, electromyography (EMG) from the neck muscles was conducted to monitor the animals’ movements. During the recordings, the mice were placed on a stationary platform in all the experiments to increase the probability of occurrence of motionless periods during which ripples would emerge. In general, the animals did not move right before the ripples; however, they sometimes tend to move after ripples occurred (Figure 1—figure supplement 1). Hence, to remove the potential role of body movement on the brain optical signals, ripples with EMG tone around them (±500 ms) were excluded (0.2847±0.1576; mean proportion ±std; n=19 animals). Then, to extract the overall neocortical activity around ripples, the activity, captured by the three modalities, was aligned with respect to the timestamp of the ripple centers (largest trough of ripples) and averaged (Figure 1c). In addition, to evaluate the coordination between hippocampal and neocortical activity, the ensemble-wise correlation coefficient of the hippocampal MUA and the activity of neocortical regions were calculated (see Methods).

**Figure 1—figure supplement 1.**
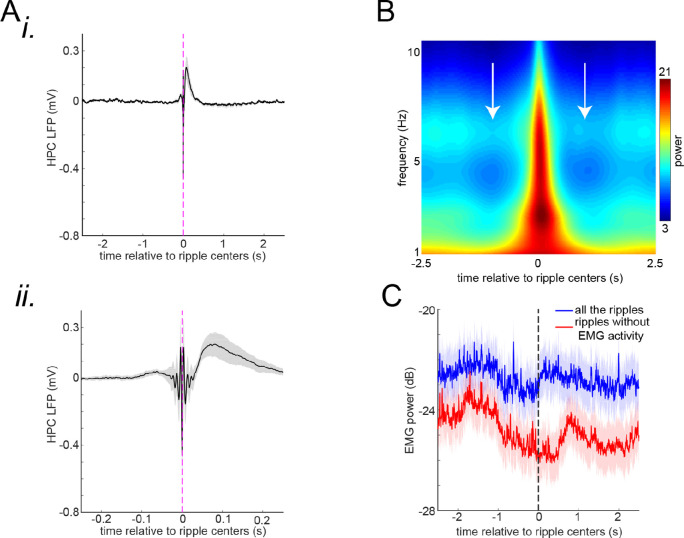
Peri-ripple theta and EMG activity. (**A**) (i) The mean peri-ripple hippocampal local field potential (HPC LFP) was first averaged in individual animals and then averaged across 25 animals. (ii) The zoomed-in version of (i). (**B**) The mean spectrogram of peri-ripple HPC LFP was first averaged in individual animals and then averaged across 25 animals. Note the high power of ~3 Hz close to 0 time corresponding to the post-ripple large deflection apparent in (**A**). Moreover, note the reduction of theta power (~6 Hz; white arrows) before and after the ripple centers time (0 time). (**C**) The mean peri-ripple EMG signal was first averaged in individual animals and then averaged across 19 animals. The blue trace is associated with all the detected ripples, and the red trace is associated with all the ripples around which (± 500 ms) no EMG activity was detected. Note that both traces show a significant reduction right before the ripple centers (0 time). Note also that the blue trace shows an elevation right after the ripple centers indicating that the animals start moving right after a proportion of ripples (0.2847±0.1576; mean±std; n=19 animals). The shadings represent the standard error of the mean.

### Population membrane voltage significantly dropped during awake ripples in the neocortical superficial layers

**Figure 2.**
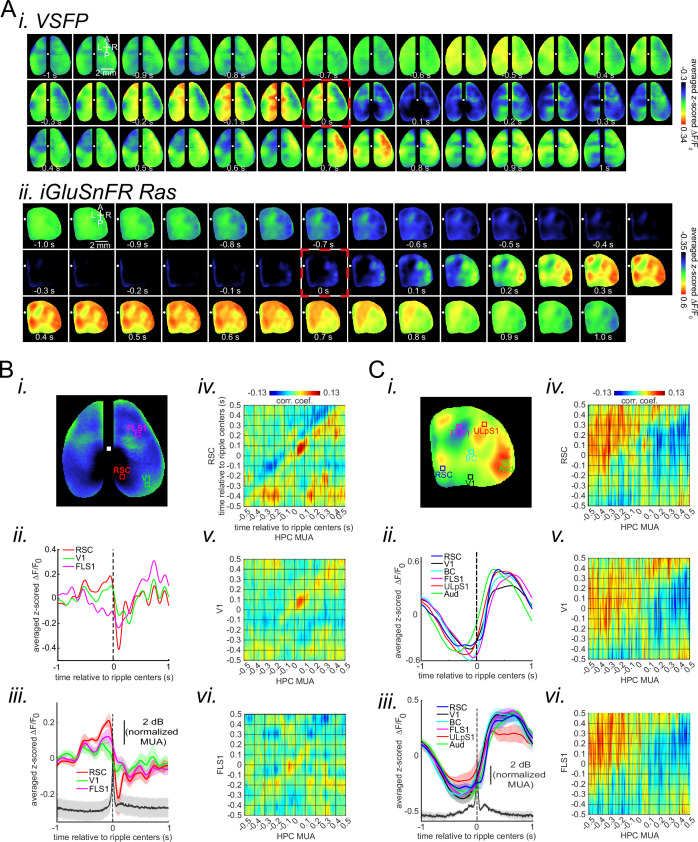
Deactivation and activation dominate the neocortical voltage and glutamate activity, respectively, during awake ripples. (**A) (i–ii**) Montage of average voltage (**i**) and glutamate (ii) activity 1 s before and after ripple centers in two representative animals. Zero time (red dashed square) represents the timestamp of the center of ripples. Note the reduction of voltage signal across neocortical regions during ripples and the elevation of voltage activity before ripples. The deactivation is the strongest in the agranular retrosplenial cortex (aRSC), the dark area in the posterior-medial part of the imaging window which is noticeable in the frame associated with the time 100 ms in (**i**). Glutamate activity, on the other hand, showed a strong activation during ripples in all the regions. (**B**) (**i–ii**) A representative frame chosen from the hyperpolarization period in (**A–i**) along with peri-ripple mean voltage time-series of three regions of interest chosen from the aRSC, primary visual cortex (**V1**), and primary forelimb somatosensory cortex (FLS1). The data represented in time-series format is the same data shown in (**A–i**). (iii) Peri-ripple mean voltage and hippocampal multi-unit activity (HPC MUA) time-series averaged across six voltage-sensitive fluorescent protein (VSFP) mice. The shading represents the standard error of the mean (SEM). aRSC shows the strongest and fastest deactivation compared with other regions. (iv–vi) Ensemble-wise correlation coefficient function of the peri-ripple voltage activity of the neocortical regions and HPC MUA. Rows and columns of the matrices represent time (in seconds) relative to ripple centers. (**C**) The same as (**B**) but for iGlu-Ras animals (n=4) with extra regions of interest from primary lip somatosensory cortex (ULpS1), primary barrel cortex (BC), and primary auditory cortex (Aud). The glutamate signal from aRSC shows the fastest and latest onset of elevation. Note the presence and absence of enhanced correlation between aRSC and HPC MUA in the time interval (0,100 ms) in the voltage and glutamate activity, respectively.

The ripple event-triggered averaged neocortical membrane voltage showed a fast hyperpolarization right after the ripple centers (Figure 2Ai, Bi-iii). These peri-ripple voltage signals in the awake state were in sharp contrast with what had been reported in sleep where a membrane depolarization dominates (Karimi Abadchi et al., 2020 Figure 2—figure supplement 1). There was significant regional variation in the hyperpolarization pattern, with aRSC showing the strongest reduction of amplitude (Figure 2B). This phenomenon was consistent in all the six animals used for this set of experiments (Figure 2—figure supplement 2A). In addition, we did not find a significant correlation between the post-ripple hyperpolarization in aRSC and ripples amplitude nor duration (Figure 2—figure supplements 5 and 6; Fernández-Ruiz et al., 2019; Nitzan et al., 2022). However, the average peri-ripple aRSC voltage activity showed a larger pre-ripple depolarization and a delayed post-ripple hyperpolarization around bundled than single ripples (Figure 2—figure supplement 6B). The pre-ripple larger depolarization might signal the occurrence of a bundled ripple (similar to the larger pre-bundled- than pre-single-ripple deactivation observed during sleep [Karimi Abadchi et al., 2020]).

**Figure 2—figure supplement 1.**
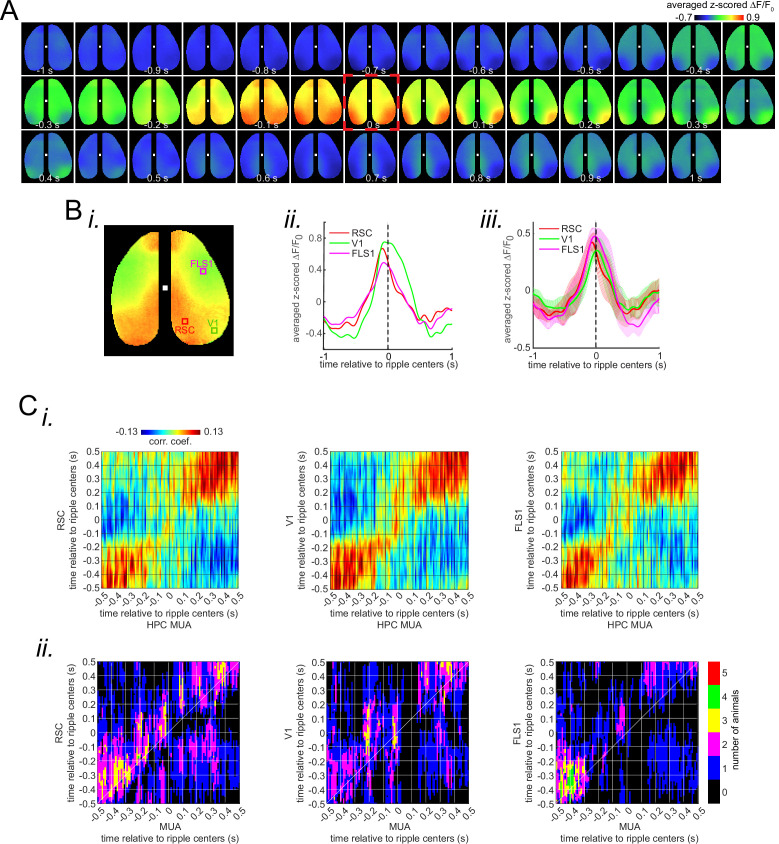
Peri-ripple voltage activity under urethane anesthesia. (**A**) Montage of average voltage activity 1 s before and after ripple centers under urethane anesthesia (as a model of sleep) in the same representative animal as in Figure 2a–i. Zero time (red dashed square) represents the timestamp of the center of ripples. Note the elevation of voltage signal (depolarization) across neocortical regions during ripples which is in sharp contrast with the result in Figure 2a–i. (**B**) (**i–ii**) A representative frame chosen from the depolarization period in (**a**) along with peri-ripple mean voltage time-series of three regions of interest chosen from the agranular retrosplenial cortex (aRSC), primary visual cortex (**V1**), and primary forelimb somatosensory cortex (FLS1). The data represented in time-series format is the same data shown in (**A**). (iii) Peri-ripple mean voltage time series under urethane anesthesia averaged across five voltage-sensitive fluorescent protein (VSFP) mice. The shading represents the SEM. (**C**) (**i**) Ensemble-wise correlation coefficient function of the peri-ripple voltage activity of the neocortical regions and hippocampal multi-unit activity (HPC MUA) recorded under urethane anesthesia (averaged across n=6 animals). Rows and columns of the matrices represent time (in seconds) relative to ripple centers. (ii) The number of VSFP animals showing significant regions in their correlation functions between peri-ripple HPC MUA and different neocortical regions based on cluster-based significant testing via temporal shuffling. The diagonal of the correlation functions is marked.

**Figure 2—figure supplement 2.**
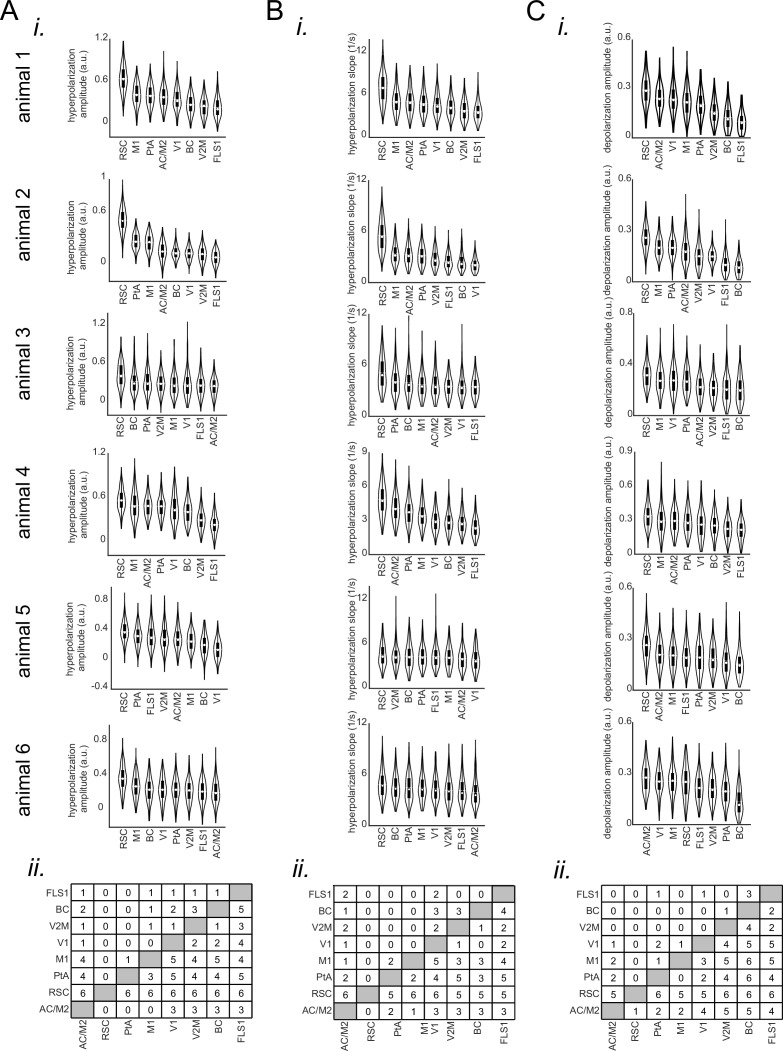
Comparing the features of the peri-ripple voltage activity across neocortical regions. (**A**) (**i**) Bootstrap distribution of voltage reduction amplitude (with respect to the pre-ripple baseline) across imaged neocortical regions for individual voltage-sensitive fluorescent protein animals. The regions are sorted according to their distribution mean in descending order (repeated-measure ANOVA with df = 7 and n=231; from top to bottom: *F*=440.597, p-value = 1.0596 × 10^–211^; *F*=858.17, p=0; *F*=62.95, p=3.0723 × 10^–46^; *F*=202.86, p=1.578 × 10^–86^; *F*=163.62, p=1.797 × 10^–82^; *F*=115.303, p=2.703 × 10^–97^). (**ii**) The result of posthoc multiple comparisons following repeated-measure ANOVA, pooled across animals (six animals). The numbers at each entry of the matrix represent the number of mice for which the bootstrap distribution associated with the corresponding row region has statistically significant larger mean than column region. Significance alpha level of 0.05 was used as the threshold for statistical significance. (**B–C**) The same as (**A**) but for voltage reduction slope (mean over full-width at half-maximum of derivative of the voltage signal; repeated-measure ANOVA with df = 7 and n=231; from top to bottom: *F*=195.096, df = 7, p-value = 1.1431 × 10^–128^; *F*=291.61, p=1.32 × 10^–161^; *F*=63.9, p=4.56 × 10^–55^; *F*=240.13, p=1.36 × 10^–146^; *F*=7.35, p=4.06 × 10^–6^; *F*=20.24, p=1.43 × 10^–20^) and pre-ripple voltage elevation amplitude (mean over full-width at half-maximum; repeated-measure ANOVA with df = 7 and n=231; from top to bottom: *F*=288.5488, df = 7, p-value = 1.8389 × 10^–142^; *F*=316.86, p=1.79 × 10^–213^; *F*=92.71, p=4.53 × 10^–74^; *F*=62.03, p=4.88 × 10^–37^; *F*=58.77, p=2.3 × 10^–50^; *F*=134.77, p=2.37 × 10^–123^). Note that agranular retrosplenial cortex (aRSC), compared with all other imaged regions, shows largest voltage reduction amplitude, fastest rate of change of voltage reduction, and largest pre-ripple voltage elevation in at least five out of six animals.

**Figure 2—figure supplement 3.**
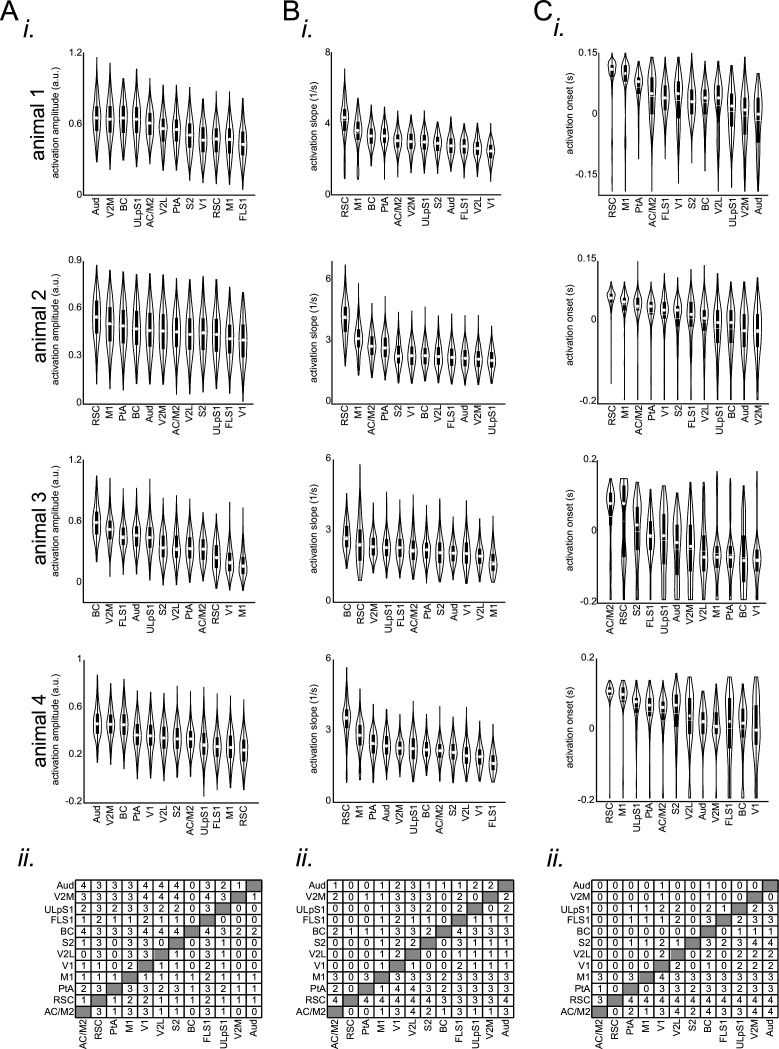
Comparing the features of the peri-ripple glutamate activity across neocortical regions. (**A**) (**i**) Bootstrap distribution of glutamate activation amplitude (with respect to the pre-ripple baseline) across imaged neocortical regions for individual iGlu-Ras animals. The regions are sorted according to their distribution mean in descending order (repeated-measure ANOVA with df = 11 and n=270; from top to bottom: *F*=337.9781, df = 11, p-value = 3.6841 × 10^–262^; *F*=304.206, p=1.3 × 10^–211^; *F*=663.42, p=0; *F*=394.13, p=4.42 × 10^–256^). (**ii**) The result of post hoc multiple comparisons following repeated measure ANOVA, pooled across animals (four animals). The numbers at each entry of the matrix represent the number of mice for which the bootstrap distribution associated with the corresponding row region has a statistically significant larger mean than the column region. The significance alpha level of 0.05 was used as the threshold for statistical significance. (**B–C**) The same as (**A**) but for glutamate activation slope (mean over full-width at half-maximum of derivative of the glutamate signal repeated-measure ANOVA with df = 11 and n=270; from top to bottom: *F*=207.5245, *P*-value = 3.427 × 10^–130^; *F*=889.69, *P*=0; *F*=81.93, *P*=2.83 × 10^–76^; *F*=249.77, *P*=1.35 × 10^–180^) and glutamate activation onset (timestamp at which the derivative of iGluSnFR signal reaches its half-maximum for the first time; repeated-measure ANOVA with df = 11 and n=270; from top to bottom: *F*=113.0048, p-value = 7.705 × 10^–149^; *F*=142.93, p=3.53 × 10^–187^; *F*=86.56, p=2.53 × 10^–119^; *F*=57.1, p=5.78 × 10^–80^). Note that agranular retrosplenial cortex (aRSC), compared with all other imaged regions, shows the fastest rate of change and the latest onset of glutamate activation in at least three out of four animals.

**Figure 2—figure supplement 4.**
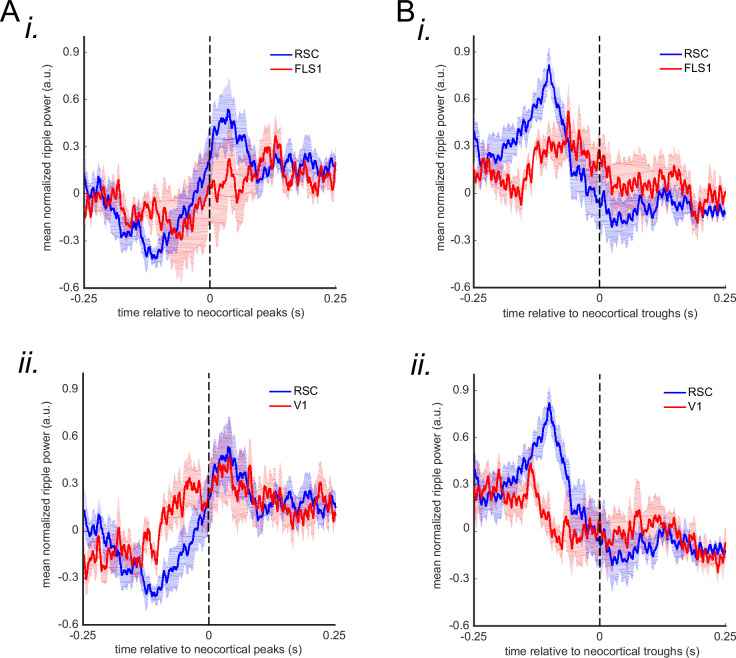
Mean ripple power triggered by peaks (**A**) and troughs (**B**) of the voltage signal captured from agranular retrosplenial cortex, V1, and forelimb somatosensory cortex 1. Zero time represents the timestamp of the neocortical troughs/peaks. The shading represents SEM (n=6 animals).

**Figure 2—figure supplement 5.**
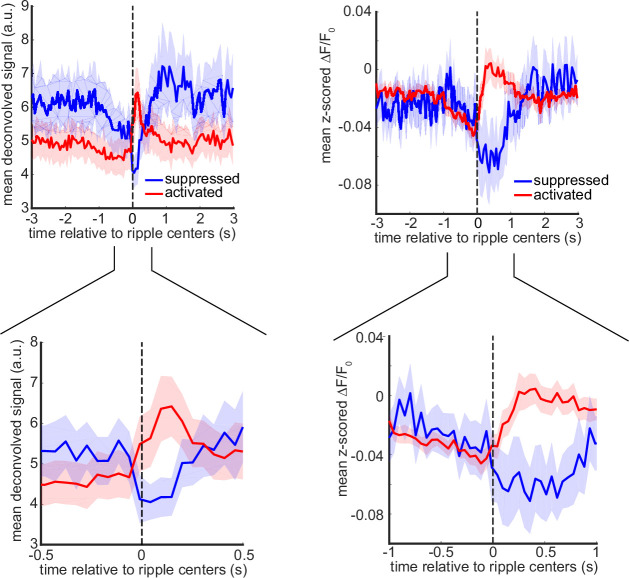
The relationship between the ripple power and the post-ripple voltage drop amplitude. (**A**) (**i**) The ripples were divided into eight subgroups using eight quantiles of their power distribution. Peri-ripple agranular retrosplenial cortex (aRSC) voltage trace was triggered on ripple centers in the odd-numbered ripple power subgroups for each animal and then averaged across six animals. The standard errors of the mean were not shown for the sake of simplicity. (**ii**) The same as the (**i**) panel but for only the lowest and highest power subgroups. The shading represents the standard error of the mean. (**B**) The scatter plots of the ripple power and pre-ripple aRSC voltage amplitude for individual animals. The black lines in each graph represent the linear regression line. The blue circles in each graph are associated with one ripple. The Pearson’s correlation values (ρ) and the p-value of their corresponding statistical significance are represented on top of each graph. (**C**) The same as (**B**) graphs but for post-ripple aRSC amplitude. To check that the correlation results were not influenced by the extreme values of the ripple power and aRSC voltage, we repeated the same correlation analysis after removing the ripples associated with top and bottom 5% of the ripple power and aRSC voltage values. According to this analysis, one out of six animals showed a negative correlation (ρ=–0.13), and five animals did not show a statistically significant correlation (p-value >0.05) between ripple power and pre-ripple aRSC voltage amplitude. Moreover, two out of six animals showed a negative correlation (same animals that showed a negative correlation before removing the extreme values; ρ=–0.12 and –0.14), one animal showed a positive correlation (ρ=0.1), and three animals did not show a statistically significant correlation (p-value >0.05) between ripple power and post-ripple aRSC voltage amplitude.

**Figure 2—figure supplement 6.**
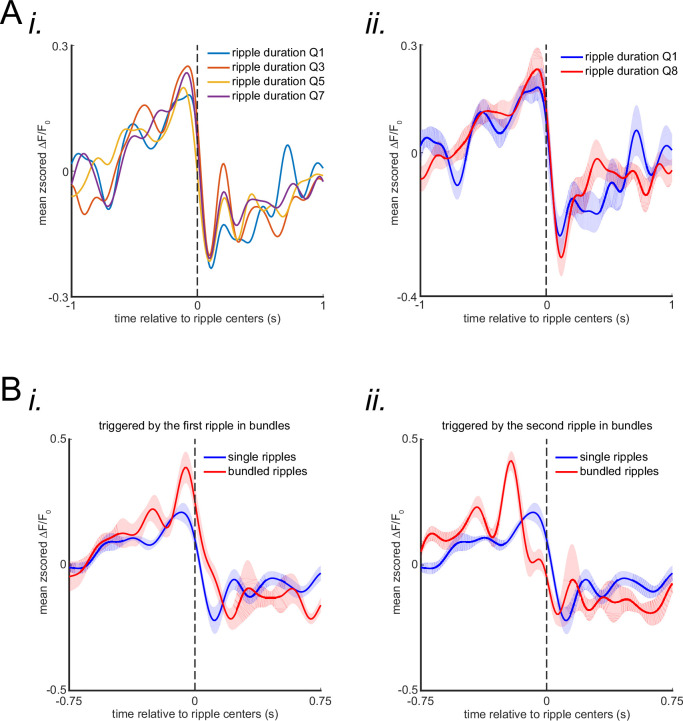
The relationship between the ripple duration and the post-ripple voltage drop amplitude. (**A**) (**i**) The ripples were divided into eight subgroups using eight quantiles of their duration distribution. Peri-ripple agranular retrosplenial cortex (aRSC) voltage trace was triggered on ripples in the odd-numbered ripple duration subgroups for each animal and then averaged across six animals. The standard errors of the mean were not shown for the sake of simplicity. (ii) The same as the (**i**) panel but for only lower and highest duration subgroups. The shading represents the standard error of the mean. (**B**) (**i**) The animal-wise average of mean peri-ripple aRSC voltage trace triggered by centers of the single and centers of the first ripple in the bundled ripples. (ii) Same as (**i**) but triggered by the centers of the second ripple in the bundled ripples.

**Figure 2—figure supplement 7.**
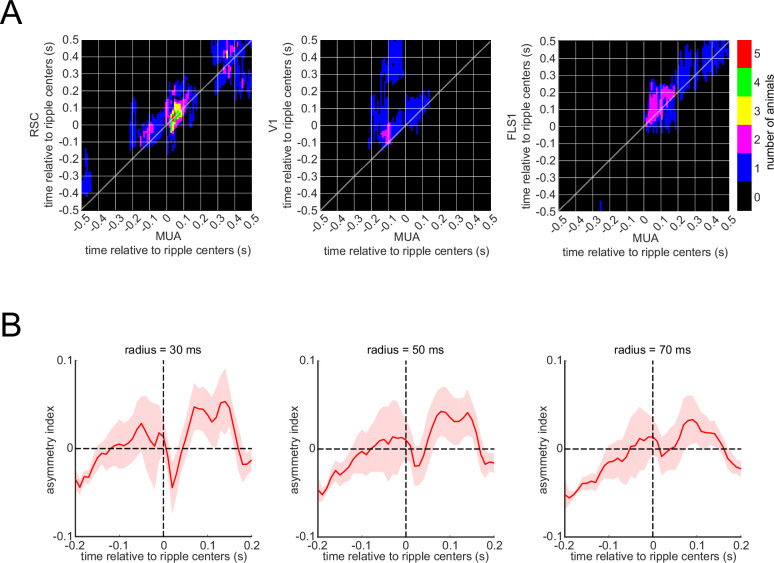
The statistical significant testing of the values of the correlation function. (**A**) The number of voltage-sensitive fluorescent protein animals showing significant regions in their correlation functions between peri-ripple hippocampal multi-unit activity (MUA) and different neocortical regions based on cluster-based significant testing via temporal shuffling. The diagonal of the correlation functions is marked. (**B**) The asymmetry index of the ensemble-wise correlation function was calculated for each time point t relative to the ripple times (t=0) for each animal and then averaged across six animals. The shading represents the standard error of the mean. The index at time t equals the average correlation value from (t − r, t+r) to (**t, t**) minus the average correlation value from (**t, t**) to (t+r, t − r) where r varies from 0 to a radius value mentioned on the top of each graph. According to this definition, a positive asymmetry index value means the hippocampal leads the neocortex, and a negative value means otherwise.

The rate of reduction of voltage was fastest in aRSC in five out of six of the animals (Figure 2—figure supplement 2B). Moreover, we observed a pre-ripple elevation of voltage which was also strongest in aRSC in five out of six animals (Figure 2Biii; Figure 2—figure supplement 2C). Lastly, the ensemble-wise correlation coefficients averaged across VSFP animals revealed a period (~0–100 ms) of enhanced coordination between hippocampal MUA and aRSC voltage activity which was absent for the somatosensory regions (Figure 2Biv-vi; Figure 2—figure supplement 7). Importantly, a fine-scale (<70 ms; Figure 2—figure supplement 7B) temporal lead/lag relationship should not be inferred from these results mainly because the temporal resolution of the two signals, one electrophysiological, and one optical, are not the same.

### Glutamate concentration increases after awake ripples in the neocortical superficial layers

Next, we performed peri-ripple averaging of glutamate indicator (iGluSnFR) signals of the neocortical superficial layers. In all imaged neocortical regions, the glutamate signal was reduced before the ripple peak (Figure 2Aii, Ci-iii). This reduction is probably associated with a brain state (i.e., quiet wakefulness) which is conducive for emergence of the ripples (Figure 1—figure supplement 1). On the other hand, after the ripples occurred, the glutamate signal increased (Figure 2Aii, Ci-iii). The amplitude of the signal varied between regions with barrel cortex (BC), primary auditory cortex (Aud), and secondary medial visual cortex (V2M) showing the highest increase while aRSC showing one of the lowest increases in the majority of the animals (Figure 2—figure supplement 3A). There was also a region-dependency in the rate (i.e., slope or derivative) and onset of glutamate concentration change with aRSC showing the steepest slope and the latest elevation onset compared with other imaged regions, especially with BC, Aud, and V2M which showed the lowest slope (Figure 2—figure supplement 3B) and the earliest onset time in the majority of the animals (Figure 2—figure supplement 3C). Lastly, the ensemble-wise correlation coefficients averaged across iGluSnFR-Ras animals did not reveal a period of enhanced coordination between hippocampal MUA and aRSC glutamate activity in close vicinity of the ripples (Figure 2Civ-vi; compare with Figure 2Biv).

### aRSC neurons show opposite patterns of peri-ripple modulation

**Figure 3.**
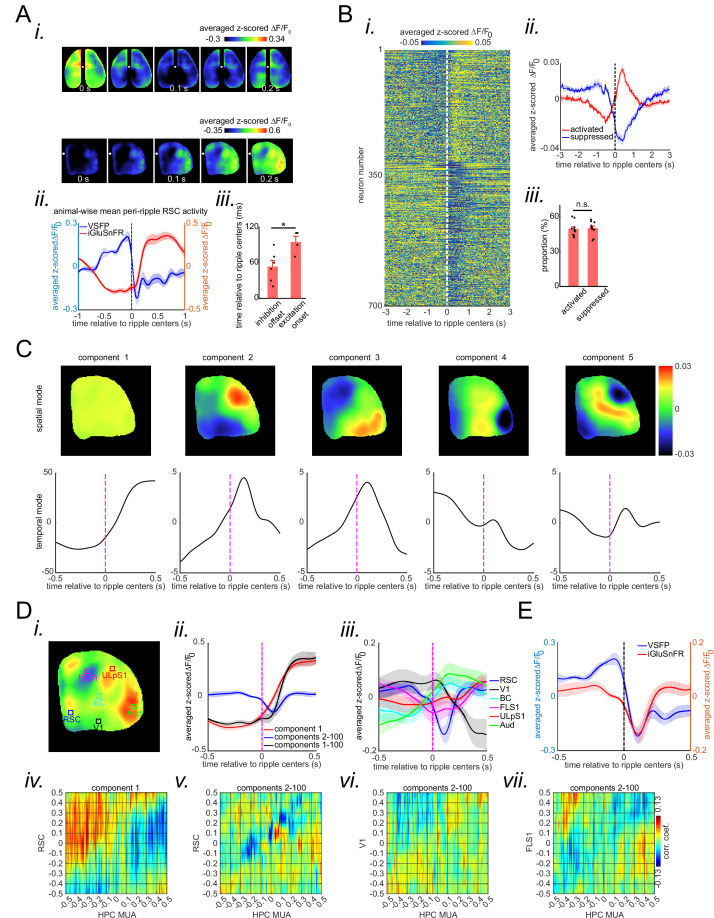
A subpopulation of agranular retrosplenial cortex (aRSC) neurons fire during awake ripples despite the strong voltage reduction. (**A**) (**i**) Five frames taken from the montages shown in Figure 2ai–ii aligned with respect to the ripple center timestamps (zero time). Note the elevation of the glutamate signal as voltage suppression eases. Also, note that voltage reduction is strongest and glutamate activation onset is the latest in aRSC compared with other regions. (**ii**) Time-series representation of the aRSC voltage (blue) and glutamate (red) signals shown in (**i**). Note that the onset of glutamate activation is around the offset of voltage suppression. (iii) Statistical comparison of the voltage suppression offset time in voltage-sensitive fluorescent protein (VSFP) mice (n=6) and glutamate onset time in iGlu-Ras mice (n=4). There is a statistically significant difference between the two (two-sample t-test; p=0.02). (**B**) (**i**) Average calcium trace (ΔF/F_0_) for individual neurons 3 s before and after ripple centers in a representative Thy1-GCamp animal. The neurons’ calcium traces are grouped into two clusters and are sorted based on their cluster membership. During ripples, the neurons in clusters 1 and 2 show elevation and suppression of calcium signal, respectively. (ii) Peri-ripple calcium traces are averaged across neurons in each cluster in each animal and then averaged across 11 animals. The shading represents the standard error of the animal-wise mean. (iii) Statistical comparison of the proportion of neurons in clusters 1 (activated) and 2 (suppressed). There is no significant difference between the two proportions (paired t-test; p>0.05). Comparing the results in (**A–ii**) and (**B–ii**) suggests that the majority of neurons in clusters 1 and 2 are likely modulated by the excitatory and inhibitory forces applied to aRSC, respectively. (**C**) Spatial and temporal modes associated with the first five largest singular values (components) of the concatenated stack of peri-ripple iGluSnFR activity in the representative iGlu-Ras animal presented in Figure 2aii. Note that the spatial mode of the first component does not show a specific topography, and the corresponding temporal mode is dominated by post-ripple elevation of the iGluSnFR signal. Also, the amplitude of the first component temporal mode is an order of magnitude larger than that in other components. (**D**) (**i**) A representative frame chosen from (**A**) with six regions of interest (ROIs) chosen from six different neocortical regions. (ii) Three animal-wise (n=4) averages of the reconstructed mean peri-ripple glutamate signals captured from the aRSC ROI in (**D-i**). The signals were reconstructed using first (red), second-to-hundredth (blue), and first-to-hundredth (black) components in (**C**). The black signal is the summation of the red and blue ones. Note that the red signal (first component) captured almost all of the elevation seen in the black signal while the blue signal (2–100 components) shows a post-ripple dip. (iii) Animal-wise average of the reconstructed mean peri-ripple glutamate signals captured from all the ROIs in (**D-i**) color-coded according to the ROIs. The signals were reconstructed using second-to-hundredth components. Note that only aRSC shows a post-ripple dip. (iv) Ensemble-wise correlation coefficient function of the peri-ripple aRSC glutamate activity (only first component) and hippocampal multi-unit activity (HPC MUA). Rows and columns of the matrices represent time (in seconds) relative to ripple centers. (v-vii) The same as (iv) but for glutamate activity of three regions reconstructed from components 2–100. Note the presence and absence of enhanced correlation between aRSC and HPC MUA in the time interval (0,100 ms) in (iv) and (**v**), respectively. (**E**) Animal-wise average of mean peri-ripple signals captured from aRSC in all VSFP (blue; n=6) and iGlu-Ras (red; n=4) animals. The signals in iGlu-Ras animals were reconstructed from 2–100 components. Note that the timing of the dips in both signals matches, suggesting they both represent the same phenomenon.

Due to the hyperpolarization of membrane potential in superficial layers of aRSC and the delayed glutamate elevation in the region (Figure 2—figure supplement 3C the onset time of RSC activation is larger than zero; Figure 3A), aRSC neurons may not fire at all during awake ripples. To address this question, we performed two-photon calcium imaging of the neurons in layers II/III of aRSC in Thy1-GCamp mice, despite the fact that the calcium signal is biased toward the burst of and not individual spikes (Huang et al., 2021). Peri-ripple averaging of single-cell calcium traces was performed, and the average traces of neurons over the interval −500 ms to +500 ms were clustered into two clusters using the k-means algorithm with correlation coefficient as the similarity metric. Second was the optimum number of clusters according to the silhouette and Calinski-Harabasz criteria. This analysis revealed that there are at least two equally sized subpopulations of neurons in aRSC; one whose calcium activity is elevated and one whose calcium activity is suppressed during and right after awake ripples (Figure 3Bi). Notably, in the ~1 s-long interval before the ripple centers, the calcium activity of elevated and suppressed sub-populations was suppressed and elevated, respectively (Figure 3Bii). The pre-ripple modulation of the two sub-populations is consistent with the excitatory and inhibitory ramps observed in Chambers et al., 2022. These results show that, despite the presence of significant reduction in population membrane voltage in aRSC, a substantially large sub-population of neurons increases their calcium activity during awake ripples. However, the timing of their calcium activity does not match that of the elevation in glutamate signal (increase in calcium precedes glutamate signal elevation). To make our results more comparable with those of electrophysiological studies, we deconvolved the calcium traces and tested for the significance of the modulation of each neuron by comparing its mean peri-ripple deconvolved trace with a neuron-specific shuffled distribution (see the Methods section for details). We found 8.46%±3 (mean ± std across 11 mice) of neurons were significantly modulated over the interval (0, 200 ms), 81.08%±8.91 (mean ± std across 11 mice) of which were up-modulated, and the rest were down-modulated (Figure 3—figure supplement 1). The observation that the elevation of calcium signal in up-modulated (activated) sub-population precedes that of glutamate activity in aRSC led us to ask whether the observed glutamate signal consists of components whose timings match those of the observed reduction in the voltage signal and the increase in the calcium activity. To address this question, we performed singular-value decomposition on the concatenated stack of individual peri-ripple glutamate activity chunks. This method decomposed the stack into components with specific spatial (Figure 3C upper row) and temporal modes. Then, for each component, the corresponding temporal mode was chunked around individual ripples, aligned, and averaged (Figure 3C lower row). Notably, the first component showed a global post-ripple elevation of glutamate activity whose amplitude was an order of magnitude larger than that in other components and explained 83.11%±6.75 (mean ± std across four iGluSnFR Ras mice). Other components, on the other hand, showed a mixture of elevation and reduction across neocortical regions. These patterns were similar across all the animals. We combined all the components with mixed patterns of modulation (components 2–100), explaining the rest of the remaining variance in the data (~17%), and reconstructed the mean peri-ripple glutamate activity across neocortical regions (Figure 3Di–iii). We observed that the mean peri-ripple glutamate activity in aRSC was decomposed into two specific patterns of post-ripple modulation, positive (Figure 3Dii; red signal; reconstructed from component 1) and negative (blue signal; reconstructed from components 2–100). Interestingly, other regions did not show the negative pattern of modulation (Figure 3Diii). In addition, the timing of the post-ripple negatively modulated glutamate signal in aRSC matched that of the voltage activity in aRSC (Figure 3E), which suggests that one of the factors involved in the reduction of voltage in aRSC could be the reduction of endogenous and/or exogenous excitatory glutamatergic input to the region. Also, the onset time of the positively modulated glutamate signal in aRSC was earlier than that of the original signal, which matches better with the timing of aRSC neurons’ calcium activity presented in Figure 3B. Lastly, the ensemble-wise correlation coefficients averaged across iGluSnFR-Ras animals revealed a period (~0–100 ms) of enhanced coordination between hippocampal MUA and negatively modulated (but not positively modulated) aRSC glutamate activity (reconstructed from components 2–100) which was absent for sensory regions (Figure 3 Div-vii; Figure 3—figure supplement 2). These results suggest that the glutamate activity could potentially be a multiplex of separate components.

**Figure 3—figure supplement 1.**
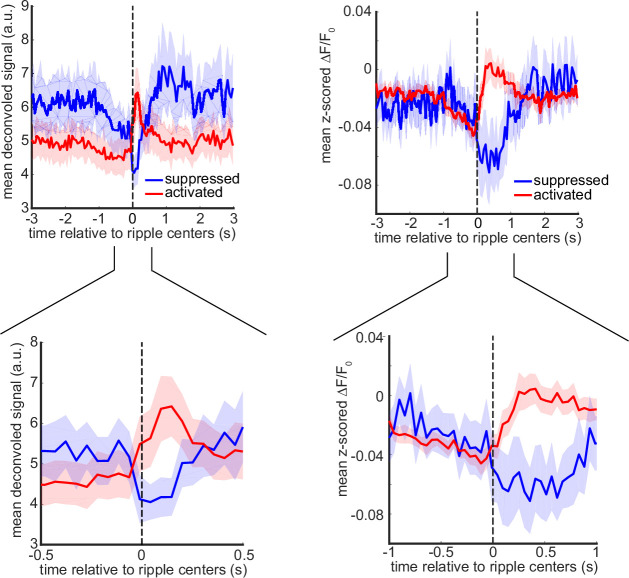
Mean peri-ripple deconvolved and calcium traces, averaged across up- or down-modulated neurons, determined based on deconvolved signal (see Methods), for each mouse and then averaged across 11 mice. The shading represents SEM.

**Figure 3—figure supplement 2.**
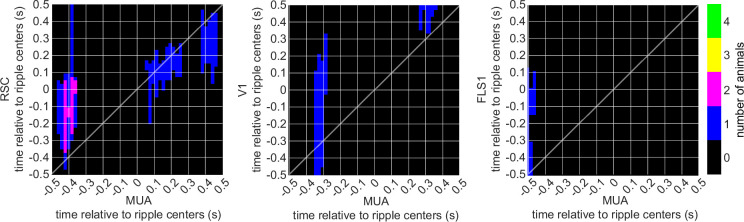
The number of iGluSnFR-Ras animals showing significant regions in their correlation functions between peri-ripple hippocampal multi-unit activity (MUA) and different neocortical regions based on cluster-based significant testing via temporal shuffling. The diagonal of the correlation functions is marked.

### The peri-ripple glutamatergic activity is layer-dependent

**Figure 4.**
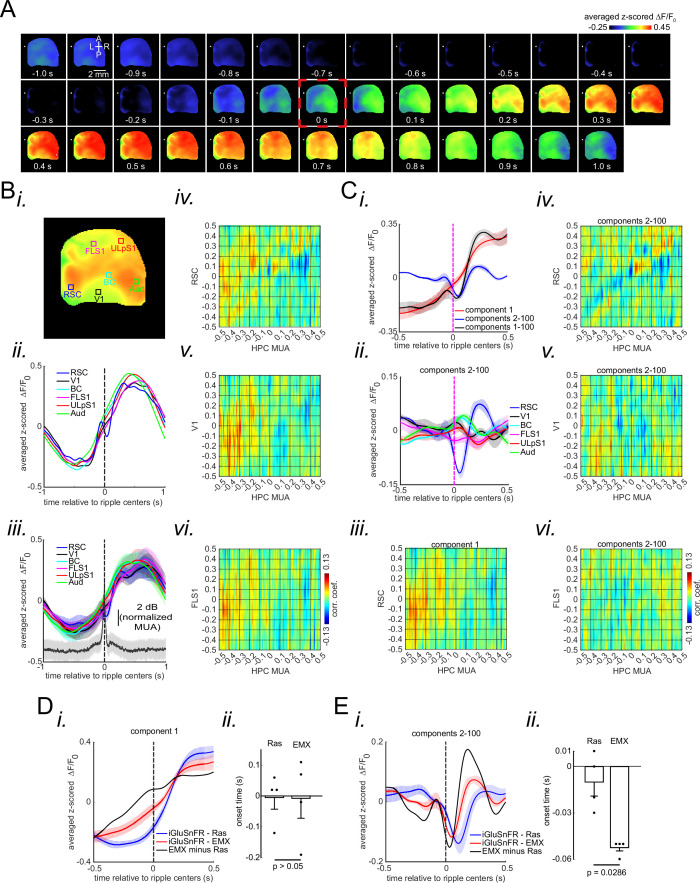
Peri-ripple glutamatergic transmission in neocortical superficial layers is delayed compared with that in deep layers. (**A**) Montage of average iGluSnFR activity 1 s before and after ripple centers in a representative iGlu-EMX animal. Zero time (red dashed square) represents the timestamp of the center of ripples. Note the elevation of glutamate signal across neocortical regions around ripple times. (**B**) (**i–ii**) A representative frame chosen from the elevation period in (**A**) along with peri-ripple mean iGluSnFR time-series of six regions of interest chosen from the agranular retrosplenial cortex (aRSC), primary visual cortex (**V1**), and primary forelimb somatosensory cortex (FLS1), primary lip somatosensory cortex (ULpS1), primary barrel cortex (BC), and primary auditory cortex (Aud). The data represented in time-series format is the same data shown in (**A**). (**iii**) Peri-ripple mean iGluSnFR and hippocampal multi-unit activity (HPC MUA) time-series averaged across four mice. The shading represents the standard error of the mean (SEM). The glutamate signals in iGlu-EMX animals are shifted to the left (precede) compared with those in iGlu-Ras animals represented in Figure 2a–ii , and c. (iv–vi) Ensemble-wise correlation coefficient function of the peri-ripple glutamate activity of the neocortical regions and HPC MUA. Rows and columns of the matrices represent time (in seconds) relative to ripple centers. (**C**) (**i**) Three animal-wise (n=4) averages of the reconstructed mean peri-ripple glutamate signals captured from the aRSC region of interest (ROI) in (**B–i**). The signals were reconstructed using first (red), second-to-hundredth (blue), and first-to-hundredth (black) components. The black signal is the summation of the red and blue ones. Note that the red signal (first component) captured almost all of the elevation seen in the black signal while the blue signal (2–100 components) shows a post-ripple dip. (ii) Animal-wise average of the reconstructed mean peri-ripple glutamate signals captured from all the ROIs in (**B–i**) color-coded according to the ROIs. The signals were reconstructed using second-to-hundredth components. Note that only aRSC shows a post-ripple dip. (iii) Ensemble-wise correlation coefficient function of the peri-ripple aRSC glutamate activity (only first component) and hippocampal HPC MUA. (iv-vi) The same as (B iv-vi) but for signals reconstructed from components 2–100. Note the presence and absence of enhanced correlation between aRSC and HPC MUA in the time interval (0,100 ms) in (iii) and (iv), respectively. (**D**) (**i**) Animal-wise (n=4) average of reconstructed (using first component) mean peri-ripple glutamate activity in iGlu-Ras (blue; n=4) and iGlu-EMX (red; n=4) animals. (**ii**) The statistical comparison of onset time in iGlu-Ras and iGlu-EMX signals in (**i**) (two-way ranksum test). (**E**) (**i**) Animal-wise average of reconstructed (using 2–100 components) mean peri-ripple glutamate activity in iGlu-Ras (blue) and iGlu-EMX (red) animals. (ii) The statistical comparison of onset time in iGlu-Ras and iGlu-EMX signals in (**i**) (two-sided ranksum test).

Given different hypothesized functions for superficial and deep layers in association with cortices (Burke et al., 2005; McNaughton, 2010), we asked whether the patterns of peri-ripple glutamate activity are layer-dependent. To address this question, we conducted wide-field optical imaging with concurrent CA1 LFP/MUA recording in EMX iGluSnFR mice with glutamate indicators expressed in excitatory neurons across all the neocortical layers (as opposed to Ras mice with only superficial layer expression). Qualitatively, the mean peri-ripple glutamate activity and ensemble-wise correlation coefficients in EMX mice (Figure 4A–B) did not differ from that in Ras mice (Figure 2Aii, C). However, the activity in EMX mice seemed to be shifted to an earlier time (compare Figure 2Ciii and Figure 4Biii). To probe the potential differences in glutamate activity in Ras and EMX mice, we compared the result of the singular value decomposition (SVD) analysis. The spatial and temporal modes associated with different components were similar in these two strains. Moreover, the reconstructed signals using first and 2–100 components showed the same pattern of positive and negative modulations, respectively (Figure 4Ci–ii). The ensemble-wise correlation coefficients averaged across iGluSnFR-EMX animals revealed a period (~0–100 ms) of enhanced coordination between hippocampal MUA and negatively modulated (but not positively modulated) aRSC glutamate activity (reconstructed from components 2–100) which was absent for sensory regions (Figure 4Ciii–vi; Figure 4—figure supplement 1).

**Figure 4—figure supplement 1.**
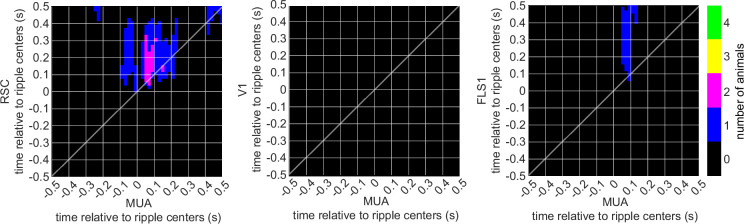
The number of iGluSnFR-EMX animals showing significant regions in their correlation functions between peri-ripple hippocampal multi-unit activity (MUA) and different neocortical regions based on cluster-based significant testing via temporal shuffling. The diagonal of the correlation functions is marked.

Moreover, we did not find a statistically significant difference in the amplitude and slope of neither positively nor negatively modulated signals between Ras and EMX groups. Additionally, although the animal-wise average of positively modulated aRSC signal showed earlier onset time (Figure 4Di), the statistical comparison between the two groups did not reach the significance threshold of 0.05 (Figure 4Dii). However, the onset time of negatively modulated signal in aRSC was significantly earlier in EMX than that in Ras group (Figure 4E). We also estimated the glutamate activity of the deep layers of aRSC by subtracting a scaled version of the Ras from the EMX signal (the black trace in Figure 4D–Ei; b $lacktrace=\frac{redtrace–0.5*bluetrace}{0.5}$). The scaling was performed to accommodate for the potential amount of variance in the superficial and deep layers explained in the EMX signal (Newton et al., 2021). The estimated positively and negatively modulated glutamate signals from the deep layers of aRSC showed a shift to an earlier time (to the left). Furthermore, the estimated negatively modulated signal (black trace in Figure 4Ei) showed a noticeable rebound with a peak around 200 ms after the ripple centers. All in all, these results suggest that deep neocortical layers in aRSC receive glutamatergic modulation earlier than superficial layers do.

## Discussion

In this study, we investigated the peri-ripple activity of the neocortex during the awake state. We utilized cell-type-specific glutamate, voltage, and calcium imaging to characterize the interactions of glutamate fluctuations, membrane potential, and calcium activity (as a proxy of the spiking activity) in excitatory neurons in several neocortical regions, especially in aRSC. In this context, synaptic input (glutamate) and membrane voltage fluctuations are considered from the perspective of the dendritic trees of either layer II-III (Ras) or layer II-III and V (EMX), occupying the majority of the volume of the superficial layers of the neocortex (Chemla and Chavane, 2010b; Chemla and Chavane, 2010a). Thus, even self-excitation (i.e. the excitation of the dendrites of a region by the axonal projections originating from the same region) is seen as input in this framework. Output is considered from the perspective of spiking-related Ca2^+^ activity in the superficial pyramidal cells.

A major observation was a reduction in post-awake-SWR membrane voltage of pyramidal cells with the strongest and fastest hyperpolarization in aRSC. The reduction of membrane voltage could be due to a reduction of the glutamatergic drive, an increase in gabaergic inhibition, or occurrence of a local awake-down-state after a portion of ripples (Luczak et al., 2013; McGinley et al., 2015; Petersen et al., 2003). We monitored the glutamate delivered to pyramidal cells to test for the first possibility. The mean peri-awake-SWR glutamate concentration did not show a reduction and instead showed a delayed elevation. Three possibilities could explain the delayed elevation of the glutamate signal in aRSC: (1) glutamate is released from pre-synaptic terminals with a delay with respect to the ripple times, (2) glutamate is pre-synaptically released on time but post-synaptically sensed with a delay with respect to the ripple times (~75 ms; Figure 3Aii–iii), and (3) glutamate is released and sensed with no delay from pre- and post-synaptic, respectively, but the glutamate signal is a mixture (e.g. additive) of components with different patterns of modulation. The first possibility is unlikely given the observed pre-ripple elevation of calcium transient in a proportion of aRSC neurons (Figure 3B). The second option is likely but with a delay in the order of ~15 ms not ~75 ms as the rise-to-half-peak-time of the iGluSnFR sensor is ~15 ms (Marvin et al., 2013). Therefore, the third option is the most probable one given that the glutamate activity, imaged in the regions other than aRSC, begins to elevate during or before the ripple times (Figure 2Ciii; Figure 2—figure supplement 3C), and thus the same phenomenon could occur in aRSC as well.

To test whether the third option could be realized in the brain, SVD analysis was performed, and it revealed two patterns of modulation, that is, negatively and positively modulated components. The motivation for investigating the idea of multiplexed glutamate transmission was further fueled by the fact that a significant subpopulation of neurons in aRSC increased their calcium activity despite the significant reduction in voltage as well as seemingly delayed (relative to the SWR centers) glutamate activity in the region. SVD decomposed the potentially multiplexed glutamate signal in aRSC and recovered two main patterns of modulation. Notably, the timing of these two patterns matched those of voltage reduction and neural firing (measured by calcium activity) in aRSC. Hence, the results of the SVD analysis provide proof of principle for the feasibility of the third option. It is worthy of note that SVD is agnostic to excitation and inhibition, and it simply captures the maximum amount of variance in the data. Thus, the two patterns of modulations, which resulted from the application of SVD, may or may not reflect brain mechanisms. However, the coincidence of the timing of these two patterns with those of the voltage reduction and calcium activity in aRSC suggests that the SVD output, to some extent, reflects truly different neuronal processes (e.g. see Yamawaki et al., 2019a).

Another possible factor in post-awake-ripple voltage reduction could be that a proportion of hippocampal ripples occurs before neocortical awake down-states. To test this hypothesis, we triggered the ripple power signal by the troughs (as proxies of the awake down-states) and peaks (as proxies of the awake up-states) of the voltage signals, captured from different neocortical regions, during periods of high ripple activity when the probability of neocortical synchronization is highest (McGinley et al., 2015; Nitzan et al., 2020). According to this analysis, the ripple power was, on average, highest before troughs of aRSC voltage signal than before those of other regions (Figure 2—figure supplement 4). On the other hand, the ripple power, on average, was not higher after the peaks of aRSC voltage signal than after those of other regions (Figure 2—figure supplement 4). This observation supports the hypothesis that a local awake down-state could occur in aRSC after occurrence of a portion of hippocampal ripples. However, a recent work (Chambers et al., 2022) reported that, out of 33 aRSC neurons whose membrane potentials were recorded, only 1 showed up-/down-states transitions (bimodal membrane potential distribution). Still, a portion (10 out of 30) of the remaining unimodal neurons showed an abrupt post-ripple hyperpolarization. In addition, they reported a modest post-ripple modulation of aRSC neurons’ membrane potential (~20% of the up/down-states transition range). Hence, these results suggest that the post-ripple aRSC hyperpolarization is not necessarily the result of the awake-down-states in aRSC.

The elevated correlation between the peri-ripple hippocampal MUA and aRSC voltage activity, seen in the region (0, 100) × (0, 100) ms of the correlation function (Figure 2Biv), was an interesting observation. One possible way of interpreting this result comes as follows. Let *x_1_(t*) and *x_2_(t*) be two random processes (RPs) with mean functions *m_1_(t*) and *m_2_(t*), representing the peri-ripple activity in two brain areas. Note that mean functions are not RPs. It could be assumed that *x_1_(t*) and *x_2_(t*) would be modeled as *x_1_(t)=m_1_(t)+e_1_(t*) and *x_2_1=m_2_(t)+e_2_(t*) where *e_1_* and *e_2_* are two RPs with zero mean at any time point, capturing the ripple-to-ripple variability of the peri-ripple activity in the respective region. For any given time point *2=t_0_*, *x_1_(t_0_*), *x_2_(t_0_*), *e_1_(t_0_*), and *e_2_(t_0_*) are random variables, and therefore, it makes sense to calculate the correlation coefficient between *x_1_(t_1_*) and *x_2_(t_2_*) for any pair of time points (*t_1_, t_2_*). It is straightforward to check that the correlation coefficient of *x_1_(t_1_*) and *x_2_(t_2_),* which is called ensemble-wise correlation at (*t_1_, t_2_*) in this work, is the same as that of *e_1_(t_1_*) and *e_2_(t_2_*). Therefore, the ensemble-wise correlation represents the statistical relationship between the ripple-to-ripple variability in the peri-ripple activity of two brain areas. That is why it is sometimes called “noise correlation” (Abbott and Dayan, 1999; Zohary et al., 1994). The existence of a significant noise correlation between peri-ripple activities of two brain areas can stem from different underlying factors including direct synaptic interactions between the two areas, having a common input from a third area (Shadlen and Newsome, 1998), and the influence of the brain global fluctuations (Ecker et al., 2014) like the slow-wave activity.

Our results show that (1) there is a significant noise correlation in the region (0, 100) × (0, 100) ms of the correlation function between the peri-ripple hippocampal MUA and aRSC (and not the other imaged neocortical regions’) voltage activity; (2) the correlation function peaks around the point (75, 75) ms (Figure 2Biv) when the mean activity in both aRSC and HPC MUA is decreasing (Figure 2Biii). Hence, it is plausible that a common source (e.g. the inhibitory circuitry of the hippocampus) underlies the reduction of activity in the two regions. If it was the case, it would be expected to have symmetry around the diagonal of the correlation function. It is because the diagonal of the correlation function represents the noise correlation between the two areas at the points (t_0_, t_0_) for all t_0_. Thus, a skewness around the diagonal could be interpreted as a lead/lag relation between the activities of the two areas. To check this idea, we calculated an asymmetry index (Stringer et al., 2019). This analysis revealed that HPC MUA precedes aRSC voltage activity by <70 ms in the region (0, 100) × (0, 100) ms of the correlation function (Figure 2—figure supplement 7B).

Since the observed correlation asymmetry could be due to the fact that the temporal resolution of the voltage signal is lower than that of MUA, this observation does not necessarily vote against the common input hypothesis discussed above. In addition, this result cannot necessarily be interpreted as that the hippocampus drives the reductions of aRSC voltage activity. On the other hand, as we reported an elevation of EMG activity after a portion of ripples (Figure 1—figure supplement 1), the inputs from the motion-generating/planning areas could also contribute to the correlated variability between peri-ripple HPC MUA and aRSC voltage activity.

Given the presence of slow-wave activity during sleep that coordinates hippocampal-neocortical interactions, we asked whether there is a significant noise correlation between HPC MUA and aRSC voltage activity under a sleep-like state (i.e. urethane anesthesia). Interestingly, the ensemble-wise correlation analysis did not reveal a significant correlation between the two signals (Figure 2—figure supplement 1C). The lack of noise correlation between the two signals under a sleep-like state could be due to the absence of deliberate motion under anesthesia/sleep, different hippocampal-neocortical interactions during sleep versus wakefulness, or distinct neuromodulation of the hippocampal-neocortical system by modulatory systems in awake versus sleep states.

Even though the peri-awake-SWR glutamate signal was potentially found to be a mixture of rising and falling components, the amplitude (explained variance) of the rising component was larger than that of the falling component. This suggests that the reduction of the glutamate activity in aRSC is not the only factor contributing to the reduction of the voltage signal in the region. Therefore, by exclusion, we suggest that inhibitory input to the superficial layers of aRSC plays a significant role in the reduction of the voltage signal. Moreover, since the majority of the volume of the neocortical superficial layers is filled with dendritic trees (Chemla and Chavane, 2010b; Chemla and Chavane, 2010a), dendritic inhibition is probably the major inhibitory process reflected in the reduction of peri-awake-SWR voltage activity.

Another interesting result that came out of the application of SVD on the peri-awake-SWR glutamate activity was the observation that the negatively modulated component was present only in aRSC and not in other recorded regions, while the positively modulated component was present in all the recorded regions. This observation supports the hypothesis of the presence of peri-awake-SWR inhibition in the neocortex, because, even though the recorded neocortical regions, except aRSC, did not have a negatively modulated glutamate activity component, they still showed a reduction in their voltage activity.

Peri-ripple modulation of multiple brain regions, such as mediodorsal thalamic nucleus (Yang et al., 2019), ventral tegmental area (Gomperts et al., 2015), subiculum (Chrobak and Buzsáki, 1994; Nitzan et al., 2020), medial prefrontal (Jadhav et al., 2016; Peyrache et al., 2009; Siapas and Wilson, 1998), anterior cingulate (Wang and Ikemoto, 2016), retrosplenial (Alexander et al., 2018), and entorhinal cortices Isomura et al., 2006 have been observed. Moreover, our data show that multiple neocortical regions, such as the auditory and barrel cortices, express glutamate elevation before aRSC does. Since the majority of these regions project to aRSC, it is plausible that a peri-awake-SWR excitatory input to aRSC comes indirectly from the hippocampus, or even independently from the hippocampus, through these intermediate neocortical regions. In addition, because some aRSC neurons appear to start firing before the SWR peaks, it is also plausible that self-excitation may occur in aRSC. In addition, as awake SWRs are involved in planning, and planning could lead to the initiation of movement (Diba and Buzsáki, 2007), another potential source of excitation in aRSC could stem from the subcortical structures involved in motion generation. This idea is in line with the observation that an increased EMG tone was detected right after some of the ripples in this study (Figure 1—figure supplement 1).

According to the current literature, there are at least two potential sources for the peri-ripple inhibitory inputs to aRSC. One possibility is long-range inhibitory projections emanating from CA1. Although a majority of tracing studies have been focused on the presence of such projections in the granular RSC (gRSC) (Jinno et al., 2007; Miyashita and Rockland, 2007; Yamawaki et al., 2019b), it is plausible that they also exist in the agranular RSC (aRSC). The second option is feed-forward inhibition originated from CA1 or gRSC. This mechanism has been reported in gRSC where peri-ripple hippocampal excitatory input activates, via subiculum, the inhibitory interneurons in gRSC, which leads to suppression of firing of many pyramidal neurons (Nitzan et al., 2020; Opalka et al., 2020). Similarly, a recent work found that inhibitory interneurons in superficial layers of aRSC increase their firing during awake ripples which, in turn, could suppress the activity of some excitatory neurons (Chambers et al., 2022).

Thalamus is another source of axonal projections to aRSC (Van Groen and Wyss, 1992b). However, it is less likely that thalamic projections contribute to the peri-awake-ripple aRSC activity modulation because multiple studies have observed the suppression of the majority of thalamic neurons during awake ripples (Logothetis et al., 2012; Nitzan et al., 2022; Yang et al., 2019). Moreover, peri-awake-ripple suppression of thalamic axons projecting to the first layer of aRSC is reported (Chambers et al., 2022).

Even though the elevation of neuronal firing is dominant around sleep SWRs, suppression of neuronal firing seems to be a predominant pattern of neuronal modulation around awake ripples. For instance, suppression of neuronal firing around awake hippocampal ripples has been reported in the medial prefrontal cortex (Jadhav et al., 2016) and gRSC (Nitzan et al., 2022). Both of these regions are heavily involved in mnemonic processing, especially through replaying/reactivating the neural traces associated with previous experiences. Given that the fidelity of awake replays is higher than that of sleep ones (Karlsson and Frank, 2009; Tang et al., 2017), it could be reasonably speculated that the peri-ripple inhibition plays a role in this higher fidelity. It probably does so via increasing the signal-to-noise ratio by suppressing the interference of the non-mnemonic neuronal populations while the mnemonic representations are being replayed during ripples (Jadhav et al., 2016). Feed-forward inhibition is commonly believed to provide a normalization operation in memory retrieval, restricting pyramidal cell output to those cells whose weight vectors best match the input vector (McNaughton and Morris, 1987).

Our results suggest that dendrites of deep pyramidal neurons, arborized in the superficial layers of the neocortex, receive glutamatergic modulation earlier than those of the superficial ones. However, the results do not provide a mechanistic explanation of the phenomenon. It is possible that the observed layer-dependency of the glutamatergic modulation would partially result from the heterogeneity of the excitatory as well as inhibitory neurons across aRSC layers. But, the question is how this heterogeneity may lead to the above-mentioned layer-dependency to which our data does not provide an answer. It could be speculated that the difference in the dendritic morphology and firing type of different types of RSC excitatory neurons (Yousuf et al., 2020) or the difference in connectivity of different RSC layers with other brain regions would play a role (Sugar et al., 2011; Van Groen and Wyss, 1992a; Whitesell et al., 2021). This is a complicated problem and could only be resolved by conducting experiments specifically designed to address this problem.

### Ideas and speculation

Given the coincidence of peri-ripple inhibition and the negatively modulated component of the glutamatergic activity in superficial layers of aRSC, it could be deduced that the same coincidence would exist in the deep layers as well. This possibility is supported by the observation that reduction of glutamate signal occurred earlier in deep than superficial layers. In that case, the deep layers would receive a peri-ripple inhibitory force before the superficial layers do. Since the back-projection from the hippocampus mainly targets the superficial neocortical layers (McNaughton, 2010), the difference in latency of peri-ripple modulation of deep versus superficial neocortical layers could be interpreted from the perspective of the memory indexing theory (Teyler and DiScenna, 1986) in the following way: At the time of awake ripples, the hippocampus communicates via a subspace of neural space, the mnemonic signal as a form of an index code to the superficial layers of RSC. At this time, the deep layers, containing the attributes of past episodic memories, do not receive a mnemonic excitatory drive to avoid interference with the retrieval of index code in the superficial layers (McNaughton, 2010). When the index code is retrieved, it is sent to the deep layers for the memory contents to be retrieved, and now the superficial layers are deprived of the mnemonic excitatory drive to avoid any interference with the content retrieval in deep layers. This hypothesized coordinated and sequential retrieval process in superficial and deep layers (McNaughton, 2010) might explain the higher fidelity of awake than sleep replays. This is because peri-ripple neocortical inhibition is rare during sleep which implies that the coordination of retrieval of hippocampal content and contextual codes is probably weaker during sleep compared to the awake state.

All in all, this study provides a multi-faceted and complementary understanding of the hippocampal-neocortical interactions around awake hippocampal ripples which could not have been obtained by conducting only one of the imaging modalities and inferring the other facets from it. Importantly, this study makes two predictions that could lead to future studies. The predictions are: (a) There exists a strong dendritic inhibition in the neocortex around awake hippocampal ripples. (b) The differential layer-dependent patterns of glutamatergic modulation play a role in the hypothesized distinct functions of the deep versus superficial neocortical layers in attribute representation and memory retrieval (McNaughton, 2010).

## Methods

### Animals

Six adult (>2 months old) male transgenic mice with VSFP Butterfly 1.2, expressed in excitatory neurons in neocortical layers II and III, were used for investigating membrane potential dynamics of neocortical regions around awake hippocampal ripples. These mice were generated by crossing the lines Ai78 (Jax023528) (Madisen et al., 2015), *Camk2a*-tTA (Jax007004), and *Rasgrf2*-2A^dCre^ (Jax022864).

Four adult (>2 months old) female transgenic mice with fluorescent glutamate indicator (iGluSnFR Xie et al., 2016), expressed in excitatory neurons in neocortical layers II and III, were used for investigating dynamics of excitatory synaptic input to the neocortical regions around awake hippocampal ripples. These mice were generated by crossing the lines Ai85 (Jax026260), *Camk2a*-tTA (Jax007004), and *Rasgrf2*-2A^dCre^ (Jax022864). These mice are called iGlu-Ras in this work.

Four adult (>2 months old) male transgenic mice with fluorescent glutamate indicator (iGluSnFR), expressed in excitatory neurons in all neocortical layers, were used for investigating dynamics of excitatory synaptic input to the neocortical regions around awake hippocampal ripples. These mice were generated by crossing the lines Ai85 (Jax026260), *Camk2a*-tTA (Jax007004), and *Emx1^IRES Cre^* (Jax005628). These mice are called iGlu-EMX in this work.

11 Thy1-GCaMP6s female mice with fluorescent calcium indicator, expressed in excitatory neurons across all neocortical layers, were used for investigating spiking dynamics of neurons in layers II/III of aRSC around awake hippocampal ripples.

Mice were housed in groups of 2–5 under a 12-hr light-dark cycle. Mice were given ad libitum access to water and standard laboratory mouse diet at all times. After head-plate/electrode implantation surgery, the mice were single-housed. The animal protocol (#2209) was approved by the University of Lethbridge Animal Care Committee and was in accordance with guidelines set forth by the Canadian Council for Animal Care.

### Surgeries for wide-field voltage and glutamate imaging experiments

On the days of surgery on mice, used in wide-field imaging experiments, subcutaneous injection of buprenorphine (0.5 gr/Kg) was delivered half an hour before the surgery started. Animals were then anesthetized with isoflurane (1–2% mixed in O_2_). After reaching the desired depth of anesthesia, the following steps were performed: (1) the skull skin was removed. (2) Hippocampal LFP electrode was implanted. (3) A head plate was implanted. (4) The muscles covering the lateral portion of the skull (on top of secondary somatosensory and auditory cortices) were removed. This step was performed only for the glutamate imaging experiments where a unilateral imaging window was used. This step allowed us to image activity of secondary somatosensory and auditory cortices. (5) The skull was covered with a thin and transparent layer of the metabond (Parkell, Inc). (6) The skull was covered with a glass coverslip. An additional bipolar electrode was implanted in the neck muscles for recording EMG activity. Animals were allowed to recover for 2 weeks before recordings started.

### Surgeries for two-photon calcium imaging experiments

On the days of surgery on mice, used in two-photon calcium imaging experiments, subcutaneous injection of buprenorphine (0.5 gr/Kg) was delivered half an hour before the surgery started. Animals were then anesthetized with isoflurane (1–2% mixed in O_2_). After reaching the desired depth of anesthesia, the following steps were performed: (1) the skull skin was removed. (2) A small craniotomy was performed to remove portion of the skull covering aRSC. (3) The exposed part of the brain was covered with a glass coverslip. (4) Hippocampal LFP electrode was implanted. (5) A head plate was implanted. (6) An additional bipolar electrode was implanted in the neck muscles for recording EMG activity. Animals were allowed to recover for 2 weeks before recordings started.

### Hippocampal LFP recording

Teflon-coated 50-µm stainless steel wires (A-M Systems) were used to make bipolar hippocampal LFP electrodes. The two tips of the electrode were separate around 0.5 mm so that the tips could record from two different depths. To implant the electrode for wide-field imaging experiments, a hole was drilled on the right hemisphere skull about 2.6 mm lateral to the midline and tangent to the posterior side of the occipital suture. Then, the electrode was gradually lowered through the hole at an angle of 57° with respect to the vertical axis (the axis perpendicular to the surface on which the stereotaxic apparatus was sitting). The electrode signal was being continuously monitored both visually and audibly. Lowering the electrode was stopped as soon as a dramatic increase in the spiking activity was heard and observed for the second time near the calculated coordinate (angle = 57°, depth = ~1.75 mm) for the pyramidal layer of the dorsal CA1. In this way, we ensured that the upper and lower tips of the electrode were placed in and beneath the pyramidal layer of the dorsal CA1, respectively. The electrode was fixated on the skull using Krazy Glue and dental cement. For the two-photon imaging experiments, a similar electrode implantation procedure was used. The only difference was that the electrode was lowered perpendicular to the surface of the brain until it reached the pyramidal layer of the dorsal CA1. In all the experiments, the hippocampal electrodes were implanted in the right hemispheres ipsilateral to the imaging window. The electrode signals were amplified using a Grass A.C. pre-amplifier Model P511 (Artisan Technology Group, IL) and digitized using a Digidata 1440 (Molecular Device Inc, CA) or National Instruments data acquisition system.

### Glutamate imaging

Blue-light-emitting diodes (Luxeon K2, 473 nm, Quadica Developments Inc, Lethbridge, Alberta) augmented with band-pass filters (Chroma Technology Corp, 467–499 nm) were used to excite iGluSnFR indicators. The fluorescence emission from iGluSnFR was filtered with a 520–580 nm band-pass filter (Semrock, New York, NY) and collected as 12-bit images at 100 Hz using a CCD camera (1M60 Pantera, Dalsa, Waterloo, ON) and an EPIX E4DB frame grabber controlled with XCAP 3.7 imaging software (EPIX, Inc, Buffalo Grove, IL). To reduce the effect of large neocortical blood vessels in imaging quality, the lens was focused into the neocortex to a depth of ~1 mm. We also recorded the iGluSnFR signal in response to different periphery stimulations under urethane anesthesia (Karimi Abadchi et al., 2020; Mohajerani et al., 2013) to functionally map the center of the hind-limb somatosensory, fore-limb somatosensory, auditory, visual, and barrel cortices.

### Voltage imaging

Blue-light-emitting diodes (Luxeon K2, 473 nm, Quadica Developments Inc, Lethbridge, Alberta) augmented with band-pass filters (Chroma Technology Corp, 467–499 nm) were used to excite the Butterfly indicator. FF580-FDi01−25x36 dichroic mirror was used for mCitrine/mKate2 emission light separation before getting filtered using a 528–555 nm and 582–602 band-pass filters (Semrock, New York, NY), respectively. The filtered signals were collected as 12-bit images at 100 Hz using two CCD cameras (1M60 Pantera, Dalsa, Waterloo, ON) and EPIX E8 frame grabber controlled with XCAP 3.7 imaging software (EPIX, Inc, Buffalo Grove, IL). To reduce the effect of large neocortical blood vessels in imaging quality, the lens was focused into the neocortex to a depth of ~0.5 mm. We also recorded the Butterfly FRET signals in response to different periphery stimulations under urethane anesthesia to functionally map the center of the hind-limb somatosensory, fore-limb somatosensory, auditory, visual, and barrel cortices.

### Two-photon calcium imaging

Two-photon calcium imaging was conducted via a Bergamo II multi-photon microscope (THORLABS). Ti:Saphire pulsed laser (Coherent) with wavelength of 920 nm and power of ~80 mW (measured at the tissue) was used to excite the calcium indicators. Scaning of the field of view was done by Galvo-Resonant X-Y mirrors. A ×16 water-immersion objective lens (Nikon) with numerical aperture of 0.8 was used for imaging. The emitted light from calcium indicators was collected via a GaAsP photomultiplier tube (Hamamatsu). The field of view size was 835×835 µm, and frames were captured at spatial resolution of 800×800 pixels and temporal resolution of 19.6 Hz. The depths of imaging were aimed between 110 and 190 µm (layers II/III).

### Preprocessing of Butterfly (VSFP) imaging data

We followed the ratiometric procedure, described in Carandini et al., 2015, with a modification to preprocess the VSFP data and obtain an estimate of the membrane potential at each pixel. The modification was that we used time-varying quantities for $\bar{A},\bar{D},\bar{A_{e}},and\bar{D_{e}}$ by calculating trends of these signals using the local regression method. We made this adjustment because we were working with spontaneous neocortical activity recorded over a long period of time while, in the Carandini et al., 2015, peri-stimulus activity over a short interval of time (couple of seconds) was analyzed.

### Preprocessing of iGluSnFR imaging data

First, low-rank reconstruction of the stack of frames, obtained via iGluSnFR imaging, was performed by applying singular-value decomposition and taking the components with the greatest associated singular values (Mitra and Pesaran, 1999). Next, for each pixel in the imaging window, a time-varying baseline (F_0_) for the iGluSnFR signal (F) was calculated. Baseline calculation was performed by applying the *locdetrend* function in the Choronux toolbox (Mitra and Bokil, 2007) (http://chronux.org/) to fit a piecewise linear curve to the pixels’ time series using the local regression method. The calculated baseline signal (F_0_) was then subtracted from the raw signal (F), and the difference signal was divided by the baseline values at each time point (Δ*F*/*F*_0_). At the end, a band pass (0.5–6 Hz) FIR filter was applied on the Δ*F*/*F*_0_ signal for each pixel.

### Preprocessing of two-photon calcium imaging data

The preprocessing of two-photon calcium imaging data was conducted via Suite2p pipeline implemented in Python 3 (Pachitariu et al., 2017). The signals from the detected candidate neuronal regions of interest (ROIs) as well as the geometric shape of the ROIs were visually inspected to screen for non-somatic compartments. The neuropil component of the calcium traces, estimated by Suite2p, was multiplied by 0.7 and subtracted from the traces (Pachitariu et al., 2017).

### SWRs detection

The raw hippocampal LFP was down-sampled to 1 kHz, filtered between 110 and 250 Hz (ripple-band) using real-valued Morlet wavelet implemented in MATLAB (MathWorks). The ripple power signal was generated by rectifying and smoothing the ripple-band filtered signal. Smoothing was performed using a rectangular window with a length of 8 ms. SWRs were identified when the ripple power signal passed the detection threshold defined by the mean plus a multiple of its standard deviation. The numerical value of the standard deviation multiplier was adjusted manually for each animal. A lower threshold (75% of the detection threshold) was used to estimate the onset and offset of each SWR. Detected events were further screened by applying a duration threshold. The timestamp of the largest trough between the onset and offset times of each detected event is referred to as the event center. At the end, events with centers less than 50ms apart were concatenated.

### Exclusion of ripples based on EMG activity

To ensure that the peri-ripple neocortical activity was least affected by movement-related brain activity, the ripples with above-threshold EMG activity within ±500 ms were excluded from all the analyses used in this study. The exclusion threshold was manually chosen for each animal.

### MUA calculation

MUA signal was calculated from hippocampal LFP using a similar method reported before (Belitski et al., 2008). Briefly, the hippocampal LFP signal was filtered above 300 Hz, rectified and smoothed with a rectangular window with the length of ~3 ms. The resultant signal was called MUA in this work.

### Z-scoring peri-ripple neocortical activity

The z-scoring of peri-ripple traces/frames was performed against a null distribution. The null distribution was obtained from traces centered round random timestamps which did not necessarily correspond to those of ripple centers, and the random timestamps were generated by randomly permuting the intervals between the successive ripple centers. All the individual peri-ripple traces/frames were z-scored against the null distribution before being analyzed further.

### Calculating the number of bootstrap draws and sampling size

The number of bootstrap draws for voltage (i.e. 193) and for glutamate (i.e. 270) imaging data was calculated to achieve the statistical power of 0.8 at significance level of 0.05 and effect size of 0.25 for a repeated-measure ANOVA design with 8 and 12 groups (i.e. regions), respectively. The power analysis was performed using the G*Power software (Faul et al., 2007). 50 was chosen as the sampling size at each bootstrap draw. This number was chosen since the correlation coefficient between the average of the bootstrap draws and the average of the whole peri-ripple ripples plateaued around this number in all the animals.

### Calculating amplitude, slope, and onset of activation and/or deactivation

For voltage peri-ripple mean traces, to report all the quantities as a positive number, the traces were inverted by multiplying a negative one to them. Deactivation amplitude was calculated as the difference between maximum value of the trace in the interval (0,200 ms) and the baseline value. The baseline value was calculated as the mean of the trace in the interval (–200 ms, 0). To calculate the onset and offset of the deactivation, the maximum value of the derivative (rate of change) of the voltage traces in the interval (–200 ms, 200 ms) was calculated. Onset and offset of deactivation were defined as the timestamps at which the derivative signal reaches half of its maximum value before and after the maximum value timestamp, respectively. The slope of deactivation was defined as the average slope of the voltage traces between their onset and offset times. Finally, the pre-ripple amplitude of the voltage traces was calculated by averaging the non-inverted trace values between the timestamps of the half-maximum value. For glutamate peri-ripple mean traces, all the calculations are the same as those applied to the voltage imaging data except the signal inversion at the very first step was not performed.

### Calculating ensemble-wise correlation coefficient function

Peri-ripple activity of each region and peri-ripple MUA could be conceived of as RPs for which we have observed multiple realizations. The observed realizations of these two RPs could be arranged in the matrix form as A(*r*,*t*) and B(*r*,*t*), respectively, where *r* and *t* represent *r*-th ripple and *t*-th time point with respect to the *r*-th ripple center. Note that all the observed realizations are aligned with respect to the ripple centers. The cross-correlation coefficient function, C(*t_1_*,*t_2_*), between RPs A and B could be estimated by calculating the sample correlation coefficient between A(:,*t_1_*) and B(:,*t_2_*). In other words, C(*t_1_*,*t_2_*) equals the correlation coefficient between *t_1_*-th column of A and *t_2_-th* column of B.

### Clustering the aRSC calcium traces

The z-scored (against the null distribution) peri-ripple calcium traces in the interval (–500 ms, 500 ms) were further z-scored with respect to their mean and standard deviation and then fed into the k-means algorithm implemented as the built-in function *kmeans* in MATLAB (MathWorks). Correlation was chosen as the distance metrics, 1000 was the maximum number of iterations, the number of clustering replication was set to 10, and OnlinePhase functionality was on.

### Determination of the significantly ripple-modulated neurons

The method described by Jadhav et al., 2016 was adopted to determine the significantly modulated neurons around ripples. For each aRSC neuron, the averaged peri-ripple deconvolved calcium trace (true average) was calculated. Then, 1000 shuffled averages were created by circularly jittering the deconvolved trace around each ripple by a random amount (up to 1 s). To obtain a ripple-modulation measure, we calculated the squared difference between the true average and the mean of the shuffled averages in the (0, 200 ms) window. To determine the ripple-modulation significance, the ripple-modulation measure for each shuffled average was calculated in the same way that was done for the true average. If the ripple-modulation measure of the true average was greater than 95% of those of the shuffled averages, the aRSC neuron was determined to be significantly modulated. To determine if a modulated neuron was up- or down-modulated (activated or suppressed), the mean over the interval (0, 200 ms) of the true average was compared with the median over the interval (–400 ms, 0) of the true average.

### Statistical tests

All statistical tests in this study were performed using MATLAB built-in functions. Repeated-measure ANOVA with Greenhouse-Geisser correction for sphericity was performed for testing the hypothesis that there was a region-effect in any of the features (i.e. amplitude, slope, onset/offset time) of the peri-ripple traces. This analysis was followed by performing multiple comparisons with Tukey-Kramer correction. For all the two-group comparisons, two-sample two-sided t-test was used.

## Data Availability

The data used to obtain the results of this article have been deposited on Dryad and can be reached via https://doi.org/10.5061/dryad.8kprr4xrk. The following dataset was generated: Karimi AbadchiJ RezaeiZ KnopfelT McNaughtonB MohajeraniMH 2023Data for: Inhibition is a prevalent mode of activity in the neocortex around awake hippocampal ripples in miceDryad Digital Repository10.5061/dryad.8kprr4xrkPMC987657036645126

## Additional files

MDAR checklist
