# Supplementary material for: Inhibition is a prevalent mode of activity in the neocortex around awake hippocampal ripples in mice
Source: eLife. 2023 Jan 16;12:e79513. doi: 10.7554/eLife.79513 (PMC9876570; doi:10.7554/eLife.79513)
Supplement: Peer review history

# Editor report: Editor's evaluation

**DOI:** 10.7554/eLife.79513.sa0

**Authors:** Adrien Peyrache

**Affiliations:** https://ror.org/01pxwe438 McGill University Canada

This paper will be of interest to neurophysiologists and systems neuroscientists interested in memory and more specifically in hippocampo-cortical interactions. Using a combination of imaging and electrophysiological techniques, the important study characterizes neocortical activity patterns around awake hippocampal ripples. The results provide solid evidence that cortical activity seems to be dominated by inhibition around awake ripples, unlike sleep ripples, but differences between intrinsic activity and synaptic transmission highlight complex interactions in the underlying neuronal circuits and dynamics.

# Decision letter

**DOI:** 10.7554/eLife.79513.sa1

**Affiliations:** https://ror.org/01pxwe438 McGill University Canada; https://ror.org/01hvx5h04 Osaka Metropolitan University Japan

Our editorial process produces two outputs: (i) public reviews designed to be posted alongside the preprint for the benefit of readers; (ii) feedback on the manuscript for the authors, including requests for revisions, shown below. We also include an acceptance summary that explains what the editors found interesting or important about the work.

**Decision letter after peer review:**

Thank you for submitting your article "Inhibition is a prevalent mode of activity in the neocortex around awake hippocampal ripples in mice" for consideration by *eLife*. Your article has been reviewed by 3 peer reviewers, and the evaluation has been overseen by a Reviewing Editor and Laura Colgin as the Senior Editor. The following individual involved in the review of your submission has agreed to reveal their identity: Kenji Mizuseki (Reviewer #3).

The reviewers have discussed their reviews with one another, and the Reviewing Editor has drafted this to help you prepare a revised submission.

Essential revisions:

In their study, Abadchi et al. investigated neocortical activity surrounding hippocampal ripples, a population event that is instrumental for memory. Specifically, the authors have used several imaging and electrophysiological techniques to examine neural activity surrounding ripples occurring during wake. The retrosplenial cortex was most strongly modulated in membrane potential and glutamatergic transmission by awake ripples. The authors also found two groups of retrosplenial cortical neurons, whose spiking activity, measured by ca^2+^ dynamics, was suppressed and enhanced by awake ripples. While the reviewers agree that the study is of potential interest, they raised several major concerns, which are summarized below. They have agreed that the below concerns must be addressed to warrant publication of the study.

1) Voltage and glutamate signals around the time of ripples show opposite effects (Figure 2). This is surprising as this was not the case during sleep (Abadchi et al., 2020, Figure 2). In addition, the analysis of the data with SVD should be improved, as it is unclear how much variance is explained by each component. Therefore, it seems inappropriate at this point to definitely conclude that ripples are characterized by enhanced inhibition in the neocortex, especially in the retrosplenial cortex. While it is not expected that the authors collect more data (e.g. direct recording of inhibitory activity), this point must be carefully explained and clarified. See reviewer #1, point #3,7.

2) The cross-correlation analysis between hippocampal MUA and neocortical activity should be improved. First, the reported signals all have different delays between the occurrence of the events and when they are actually detected. This must be discussed (reviewer #2, point #1). Furthermore, this cross-correlation analysis lacks proper statistics and seems to indicate a result at odds with the conclusion, namely that hippocampal multiunit activity is positively correlated with cortical voltage traces following ripples. A decrease in voltage following ripples should show a negative correlation (reviewer #1, points #4-5).

3) Cortical activity should be analyzed as a function of ripple parameters, as ripples of different power, for example, may lead to different cortical responses (see reviewer #1, point #6).

4) Cortical response to ripples should be analyzed as a function of the ongoing cortical state, as during wakefulness it is known for being a continuum of desynchronized to a synchronized state. Different ongoing states may result in strikingly different responses (reviewer #1, point #1).

5) The estimation of deep pyramidal cells' glutamate activity by subtracting the Ras group (Figure 4) should be better justified. As it seems that the activity from the two different cell populations was collected in different animals, the study should present convincing evidence that potential confounds (e.g. different levels of virus expression, etc.) can be ruled out (reviewer #1, point #8; reviewer #2, point #2). Furthermore, the study should discuss the difference across layers, which could potentially result from differences in cell types (Reviewer #2, point #3) and the fact that calcium levels may not always reveal the same underlying activity (reviewer #2, point #4).

*Reviewer #2 (Recommendations for the authors):*

I suggest that the authors should do the following:

The Results sections are very descriptive. It will be very helpful to include a few sentences about the importance or the significance of the results within each section.

[Editors’ note: further revisions were suggested prior to acceptance, as described below.]

Thank you for resubmitting your work entitled "Inhibition is a prevalent mode of activity in the neocortex around awake hippocampal ripples in mice" for further consideration by *eLife*. Your revised article has been evaluated by Laura Colgin (Senior Editor) and a Reviewing Editor.

The manuscript has been improved but the changes made by the authors still seem quite superficial while the comments made by the reviewers were substantial.

Specifically, there are two remaining issues that need to be addressed, as outlined below in reviewer #1's comments (with which reviewer #2 agreed).

*Reviewer #1 (Recommendations for the authors):*

In the revised version of the manuscript, the authors have added additional analyses and improved some of the existing analyses. The paper benefited from the revision, but in my view, we see mostly a number of descriptive details that stand in contradiction.

Most importantly, the main message of this manuscript is still unclear to me: as reflected in its title, this paper concludes that inhibition is the main mode of activity in the RSC during SWR, yet 2/3 of the datasets presented (glutamate imaging and single-cell data, where >80% of significantly modulated cells were up-modulated), suggest the opposite.

Lacking a theory for their functional significance, these results are mostly of interest to SWR specialists.

1) It is still not clear to me what novel conceptual advance is provided by the ensemble-wise analysis. As the authors point out in response to my previous point #4, the strongest positive correlation between HPC MUA and RSC voltage activity is around (75,75) ms. It is interesting that the correlation is maximal after the SWR peak, but this point is not discussed and I wonder whether this is simply because of the temporal lag between the two signals. More importantly, and given the authors' statement that "the correlation between the two random processes cannot be inferred from the temporal relationship between their mean functions" I wonder whether this is the most appropriate analysis here. The authors should better motivate, interpret and discuss this analysis, or substitute it altogether with a different analysis that is sensitive to temporal aspects, such as a simple event by event-wise cross-correlation.

2) I am still uneasy with the SVD analysis and its interpretation. The authors point to the similarity between aRSC voltage activity and the summed 2-100 SVD components as evidence for the peri-SWR aRSC glutamatergic activity being a mixture of up- and down-modulated components. They further postulate that the negative components, reflecting a reduction of glutamatergic input to the aRSC, could be involved in the peri-SWR voltage reduction. However, this interpretation is unlikely given that the first principal component, which accounts for the vast majority of variance in the data (>80%) is positive (i.e., glutamatergic inputs strongly increase). I also note that, given that in nearly every area that was investigated in the context of SWR there are both up and down-modulated neurons, an arbitrary combination of PCs could result in negative patterns for other areas as well. Altogether, this part of the manuscript is the weakest in my view and seems to be based on the apparent similarity between signals of different sources and with different time lags that may or may not be incidental. In my opinion, the SVD analysis does not contribute to the manuscript in its current form. If the authors wish to retain this analysis, they should establish a firmer link between the voltage, glutamate, and 2-P signals (e.g., by establishing a ground truth based on other stimuli known to be excitatory/ inhibitory), or motivate it using a rigorous theoretical framework.

*Reviewer #2 (Recommendations for the authors):*

The authors adequately addressed all our comments.

They have decided to not take up the recommendation to switch away from the jet color scheme, explaining it is a matter of taste. While I will not press this issue, I would argue that in the case of the color map jet it is not a matter of taste, instead using jet creates misleading and falsely emphasized results (see also https://blogs.mathworks.com/headlines/2018/10/10/a-dangerous-rainbow-why-colormaps-matter/). Therefore, while I understand that changing now all figures is a lot of work, I will press the authors to at least not use jet in the future anymore.

# Reply: Author response

**DOI:** 10.7554/eLife.79513.sa2

Essential revisions:

Reviewer #2 (Recommendations for the authors):

I suggest that the authors should do the following:

The Results sections are very descriptive. It will be very helpful to include a few sentences about the importance or the significance of the results within each section.

We prefer to leave the interpretation of the results for the Discussion section.

[Editors’ note: further revisions were suggested prior to acceptance, as described below.]

Reviewer #1 (Recommendations for the authors):

In the revised version of the manuscript, the authors have added additional analyses and improved some of the existing analyses. The paper benefited from the revision, but in my view, we see mostly a number of descriptive details that stand in contradiction.

Most importantly, the main message of this manuscript is still unclear to me: as reflected in its title, this paper concludes that inhibition is the main mode of activity in the RSC during SWR, yet 2/3 of the datasets presented (glutamate imaging and single-cell data, where >80% of significantly modulated cells were up-modulated), suggest the opposite.

Lacking a theory for their functional significance, these results are mostly of interest to SWR specialists.

1) It is still not clear to me what novel conceptual advance is provided by the ensemble-wise analysis. As the authors point out in response to my previous point #4, the strongest positive correlation between HPC MUA and RSC voltage activity is around (75,75) ms. It is interesting that the correlation is maximal after the SWR peak, but this point is not discussed and I wonder whether this is simply because of the temporal lag between the two signals. More importantly, and given the authors' statement that "the correlation between the two random processes cannot be inferred from the temporal relationship between their mean functions" I wonder whether this is the most appropriate analysis here. The authors should better motivate, interpret and discuss this analysis, or substitute it altogether with a different analysis that is sensitive to temporal aspects, such as a simple event by event-wise cross-correlation.

The peri-ripple activity in given brain areas such as aRSC and hippocampus could be conceptualized as random processes (RPs) where each individual ripple is associated with a realization (or sample) of the process. According to the theory of RPs, the correlation between two processes, capturing the statistical relationship between the two, has an ensemble-wise (and not event-wise) definition (Papoulis and Pillai, 2002). That is why, in this work, we calculated the ensemble-wise correlation between the ensembles of peri-ripple hippocampal and neocortical activities.

One possible way of interpreting the result of such correlation analysis comes as follows. Let *x_1_(t)* and *χ2(t)* be two RPs with mean functions *m_1_(t)* and *m_2_(t)*, representing the peri-ripple activity in two brain areas. Note that mean functions are not RPs. It could be assumed that *x_1_(t)* and *χ2(t)* would be modeled as *x_1_(t) = m_1_(t) + e_1_(t)* and *χ2(t) = m_2_(t) + e_2_(t)* where *e_1_* and *e_2_* are two RPs with zero mean at any time point, capturing the ripple-to-ripple variability of the peri-ripple activity in the respective region. For any given time point *t = t_0_*, *x_1_(t_0_)*, *χ2(t_0_)*, *e_1_(t_0_)*, and *e_2_(t_0_)* are random variables, and therefore, it makes sense to calculate the correlation coefficient between *x_1_(t_1_)* and *χ2(t_2_)* for any pair of time points *(t_1_, t_2_)*. It is straightforward to check that the correlation coefficient of *x_1_(t_1_)* and *χ2(t_2_),* which is called ensemble-wise correlation at *(t_1_, t_2_)* in this work, is the same as that of *e_1_(t_1_)* and *e_2_(t_2_)*. Therefore, the ensemble-wise correlation represents the statistical relationship between the ripple-to-ripple variability in the peri-ripple activity of two brain areas. That is why it is sometimes called “noise correlation” (Abbott and Dayan, 1999; Zohary et al., 1994). The existence of a significant noise correlation between peri-ripple activities of two brain areas can stem from different underlying factors including direct synaptic interactions between the two areas, having a common input from a third area (Shadlen and Newsome, 1998), and the influence of the brain global fluctuations (Ecker et al., 2014) like the slow-wave activity.

Our results show that (1) there is a significant noise correlation in the region (0, 100) × (0, 100) ms of the correlation function between the peri-ripple hippocampal MUA and aRSC (and not the other imaged neocortical regions’) voltage activity; (2) the correlation function peaks around the point (75, 75) ms (Figure 2Biv) when the mean activity in both aRSC and HPC MUA are decreasing (Figure 2Biii). Hence, it is plausible that a common source (e.g., the inhibitory circuitry of the hippocampus) underlies the reduction of activity in the two regions. If it was the case, it would be expected to have symmetry around the diagonal of the correlation function. It is because the diagonal of the correlation function represents the noise correlation between the two areas at the points (t_0_, t_0_) for all t_0_. Thus, a skewness around the diagonal could be interpreted as a lead/lag relationship between the activities of the two areas. To check this idea, we calculated an asymmetry index (Stringer et al., 2019), as suggested by reviewer 1 in the previous round of the review process. As presented in Author response image 1, the analysis revealed that HPC MUA precedes aRSC voltage activity by <70 ms in the region (0, 100) × (0, 100) ms of the correlation function.

**Author response image 1.** The asymmetry index of the ensemble-wise correlation function was calculated for each time point t relative to the ripple times (t = 0) for each animal and then averaged across 6 animals. The shading represents the standard error of the mean. The index at time t equals the average correlation value from (t – r, t + r) to (t, t) minus the average correlation value from (t, t) to (t + r, t – r) where r varies from 0 to a radius value mentioned on the top of each graph. According to this definition, a positive asymmetry index value means the hippocampal leads the neocortex, and a negative value means otherwise.

Since the observed correlation asymmetry could be due to the fact that the temporal resolution of the voltage signal is lower than that of MUA, this observation does not necessarily vote against the common input hypothesis discussed above. In addition, this result cannot necessarily be interpreted as that the hippocampus drives the reductions of aRSC voltage activity. On the other hand, as we reported an elevation of EMG activity after a portion of ripples (Figure 1—figure supplement 1), the inputs from the motion-generating/planning areas could also contribute to the correlated variability between peri-ripple HPC MUA and aRSC voltage activity.

Given the presence of slow-wave activity during sleep that coordinates hippocampal-neocortical interactions, we asked whether there is a significant noise correlation between HPC MUA and aRSC voltage activity under a sleep-like state (i.e., urethane anesthesia). Interestingly, the ensemble-wise correlation analysis did not reveal a significant correlation between the two signals as presented in Author response image 2.

**Author response image 2.** (i) Ensemble-wise correlation coefficient function of the peri-ripple voltage activity of the neocortical regions and hippocampal multi-unit activity (MUA) recorded under urethane anesthesia (averaged across n = 6 animals). Rows and columns of the matrices represent time (in seconds) relative to ripple centers. (ii) The number of VSFP animals showing significant regions in their correlation functions between peri-ripple HPC MUA and different neocortical regions based on cluster-based significant testing via temporal shuffling. The diagonal of the correlation functions are marked.

The lack of correlation between the two signals under a sleep-like state could be due to the absence of deliberate motion under anesthesia/sleep, different hippocampal-neocortical interactions during sleep versus wakefulness, or distinct neuromodulation of the hippocampal-neocortical system by modulatory systems in awake versus sleep states.

Lastly, reviewer 1 had asked (1) whether the difference in temporal resolution of the MAU and voltage signals could explain the observed post-ripple noise correlation, and (2) whether an event-wise correlation analysis was more appropriate. The underlying assumption of the first question is that there is a significant correlation between the two signals at the time of ripples. Given this assumption, there must have been a significant correlation in the correlation function at the point *(0, 0),* if there was no delay between the two signals, or at the point *(0, t_0_)* for *t_0_ > 0* if there was a delay between the two signals. The left panel in Figure 2 and figure supplement 7 shows that 2 out of 6 animals showed a significant correlation at points *(0, t_0_)* for 0 < *t_0_ < 150 ms* while 5 out of 6 animals showed a significant correlation at points around *(50, 50) ms*. Thus, our data votes against the validity of the assumption. As for the second question, the event-wise correlation would be identical to the ripple-triggered average analysis if the MUA activity was a Dirac δ function at the time of ripples. It is because the Dirac δ function is the neutral element of the convolution operator, and the correlation is defined as a convolution operation. In practice, peri-ripple HPC MUA is not a δ function. However, if the time course of peri-ripple HPC MUA is much shorter than that of the aRSC voltage activity (which is relatively the case in our study), the event-wise correlation analysis should generate a result closely resembling the result of the ripple-triggered average analysis. To confirm this prediction, we calculated event-wise correlation for each individual animal and then averaged the results across animals. Author response image 3 presents the result.

**Author response image 3.** 

To address reviewer 1’s point 1, the Discussion section was expanded (lines 294-341 were added), and Figure 2—figure supplements 1 and 7 were expanded.

2) I am still uneasy with the SVD analysis and its interpretation. The authors point to the similarity between aRSC voltage activity and the summed 2-100 SVD components as evidence for the peri-SWR aRSC glutamatergic activity being a mixture of up- and down-modulated components. They further postulate that the negative components, reflecting a reduction of glutamatergic input to the aRSC, could be involved in the peri-SWR voltage reduction. However, this interpretation is unlikely given that the first principal component, which accounts for the vast majority of variance in the data (>80%) is positive (i.e., glutamatergic inputs strongly increase). I also note that, given that in nearly every area that was investigated in the context of SWR there are both up and down-modulated neurons, an arbitrary combination of PCs could result in negative patterns for other areas as well. Altogether, this part of the manuscript is the weakest in my view and seems to be based on the apparent similarity between signals of different sources and with different time lags that may or may not be incidental. In my opinion, the SVD analysis does not contribute to the manuscript in its current form. If the authors wish to retain this analysis, they should establish a firmer link between the voltage, glutamate, and 2-P signals (e.g., by establishing a ground truth based on other stimuli known to be excitatory/ inhibitory), or motivate it using a rigorous theoretical framework.

The motivation for the SVD analysis was the observations that (1) aRSC voltage activity drops during and after ripples, (2) glutamate activity starts rising at the end of voltage drop in aRSC, and (3) both raw and deconvolved calcium traces for a portion of aRSC neurons elevate during or after ripples. What drives the elevation of calcium activity if the glutamate elevation is delayed with respect to the ripple times? Note that, this question would have not been asked, had the 2P calcium imaging not been performed.

3 possibilities could explain the delayed elevation of the glutamate signal in aRSC: (1) glutamate is released from pre-synaptic terminals with a delay with respect to the ripple times, (2) glutamate is pre-synaptically released on time but post-synaptically sensed with a delay with respect to the ripple times (~ 75 ms; Figure 3Aii-iii), and (3) glutamate is released and sensed with no delay from pre- and post-synaptic respectively, but the glutamate signal is a mixture (e.g. additive) of components some of which would drive the calcium transient elevation. The first possibility is unlikely given the observed pre-ripple elevation of calcium transient in a proportion of aRSC neurons (Figure 3B). The second option is likely but with a delay in the order of ~15 ms not ~75 ms as the rise-to-half-peak-time of the iGluSnFR sensor is ~15 ms (Marvin et al., 2013). Therefore, the third option is the most probable one given that the glutamate activity, imaged in the regions other than aRSC, begins to elevate during or before the ripple times (Figure 2Ciii; Figure 2—figure supplement 3C), and thus the same phenomenon could occur in aRSC as well.

To test whether the third option could be realized in the brain, SVD analysis was performed, and it revealed that the time course of the first principal component relatively matched that of the calcium transient elevation. Interestingly, when the rest of PCs were combined, aRSC, and no other imaged regions, showed a reduction of glutamate activity whose timing matched that of the voltage reduction in aRSC. Hence the results of the SVD analysis provide proof of principle for the feasibility of the third option. The seeming dichotomy in the glutamate activity could be a reflection of the feed-forward excitation and inhibition originated from HPC and/or gRSC (Yamawaki et al., 2019); however, we cannot be certain about it given that SVD is agnostic to the brain mechanisms and only cares about capturing maximum variance. That is why we were cautious about interpreting the results of the SVD analysis and kept our interpretations speculative.

The definite resolution to the puzzling dynamic of the glutamate signaling could be provided by conducting simultaneous glutamate, voltage, and 2P calcium imaging with optogenetic stimulation of the axonal terminals ended in aRSC and/or stimulating somas of the hippocampal/subicular/gRSC neurons projecting to aRSC. This design would help establish a firm relationship between all three signals which in turn would help interpret the results better. However, conducting such an experiment is currently challenging if not impossible. It is because the optogenetic stimulation, even if it is targeted at the hippocampal somas, would saturate the glutamate and voltage sensors in aRSC for a period that is longer than the temporal window of the peri-ripple modulation of the signals.

We would also like to emphasize on the basis of our conclusion on the potential prevalence of inhibition in the neocortex around awake (and not sleep) hippocampal ripples. The conclusion was based on the observation that despite the elevation of the glutamate activity (> %80 of the variance), aRSC voltage drops. In the Discussion section, we argued that the voltage drop might not be due to the presence of the awake-up/down-states in the neocortex (lines 276-293). Thus, by exclusion, inhibition would be the primary candidate for the voltage drop. Since the superficial layers of the neocortex are mainly filled with dendritic trees, the dendritic inhibition likely shunts the excitatory effect of the elevated glutamate.

To address reviewer 1’s point 2, the Discussion section was expanded (lines 246-262 were added).

References

Abbott LF, Dayan P. 1999. The effect of correlated variability on the accuracy of a population code. *Neural Comput* **11**:91–101. doi:10.1162/089976699300016827

Ecker AS, Berens P, Cotton RJ, Subramaniyan M, Denfield GH, Cadwell CR, Smirnakis SM, Bethge M, Tolias AS. 2014. State Dependence of Noise Correlations in Macaque Primary Visual Cortex. *Neuron* **82**:235–248. doi:10.1016/J.NEURON.2014.02.006

Marvin JS, Borghuis BG, Tian L, Cichon J, Harnett MT, Akerboom J, Gordus A, Renninger SL, Chen T-W, Bargmann CI, Orger MB, Schreiter ER, Demb JB, Gan W-B, Hires SA, Looger LL. 2013. An optimized fluorescent probe for visualizing glutamate neurotransmission. *Nat Methods* **10**:162–170. doi:10.1038/nmeth.2333

Papoulis A, Pillai SU. 2002. Probability, Random Variables and Stochastic Processes, Fourth Edition, 4th ed. McGraw Hill.

Shadlen MN, Newsome WT. 1998. The Variable Discharge of Cortical Neurons: Implications for Connectivity, Computation, and Information Coding. *J Neurosci* **18**:3870–3896. doi:10.1523/JNEUROSCI.18-10-03870.1998

Stringer C, Pachitariu M, Steinmetz N, Carandini M, Harris KD. 2019. High-dimensional geometry of population responses in visual cortex. *Nature* **571**:361–365. doi:10.1038/s41586-019-1346-5

Yamawaki N, Corcoran KA, Guedea AL, Shepherd GMG, Radulovic J. 2019. Differential Contributions of Glutamatergic Hippocampal→Retrosplenial Cortical Projections to the Formation and Persistence of Context Memories. *Cereb Cortex* **29**:2728–2736. doi:10.1093/cercor/bhy142

Zohary E, Shadlen MN, Newsome WT. 1994. Correlated neuronal discharge rate and its implications for psychophysical performance. *Nat 1994 3706485* **370**:140–143. doi:10.1038/370140a0
